# Insights into Dynamics of Mobile Genetic Elements in Hyperthermophilic Environments from Five New *Thermococcus* Plasmids

**DOI:** 10.1371/journal.pone.0049044

**Published:** 2013-01-11

**Authors:** Mart Krupovic, Mathieu Gonnet, Wajdi Ben Hania, Patrick Forterre, Gaël Erauso

**Affiliations:** 1 Unité Biologie Moléculaire du Gène chez les Extrêmophiles, Département de Microbiologie, Institut Pasteur, Paris, France; 2 Laboratoire de Microbiologie des Environnements Extrêmes, UMR6197 CNRS-IFREMER-Université de Bretagne Occidentale, Plouzané, France; 3 Equipe Microbiologie Environnementale et Biotechnologie, Mediterranean Institute of Oceanography (MIO), UMR CNRS 7294/IRD 235, Marseille France; 4 Laboratoire de Biologie Moléculaire du Gène chez les Extrêmophiles Institut de Génétique et Microbiologie, CNRS-UMR 8621, Université Paris-Sud 11, Orsay, France; Universidad Miguel Hernandez, Spain

## Abstract

Mobilome of hyperthermophilic archaea dwelling in deep-sea hydrothermal vents is poorly characterized. To gain insight into genetic diversity and dynamics of mobile genetic elements in these environments we have sequenced five new plasmids from different *Thermococcus* strains that have been isolated from geographically remote hydrothermal vents. The plasmids were ascribed to two subfamilies, pTN2-like and pEXT9a-like. Gene content and phylogenetic analyses illuminated a robust connection between pTN2-like plasmids and *Pyrococcus abyssi* virus 1 (PAV1), with roughly half of the viral genome being composed of genes that have homologues in plasmids. Unexpectedly, pEXT9a-like plasmids were found to be closely related to the previously sequenced plasmid pMETVU01 from *Methanocaldococcus vulcanius* M7. Our data suggests that the latter observation is most compatible with an unprecedented horizontal transfer of a pEXT9a-like plasmid from *Thermococcales* to *Methanococcales*. Gene content analysis revealed that thermococcal plasmids encode Hfq-like proteins and toxin-antitoxin (TA) systems of two different families, VapBC and RelBE. Notably, although abundant in archaeal genomes, to our knowledge, TA and *hfq*-like genes have not been previously found in archaeal plasmids or viruses. Finally, the plasmids described here might prove to be useful in developing new genetic tools for hyperthermophiles.

## Introduction

Plasmids are extrachromosomal genetic companions of cellular organisms in all three domains of life. Together with viruses and transposons, plasmids comprise the mobilome, a totality of mobile genetic elements, which exerts a significant force on the evolution of their hosts. Plasmids are perhaps best known for their promiscuous nature and ability to promote horizontal gene transfer (HGT) in microbial populations. In addition, plasmids often encode various toxicity and restriction factors that modulate the survival and fitness of their—often medically or biotechnologically important—hosts. Consequently, much of the research on plasmids has focused on elucidation of the HGT mechanisms and development of genetic tools for manipulation of model microorganisms, typically bacteria. As a result, an in-depth insight into several bacterial plasmid systems has been achieved; the knowledge on archaeal plasmids, however, is still scarce. From ecological and evolutionary perspectives, the genetic diversity of plasmids, their evolutionary relationship to other types of mobile genetic elements, interplay between plasmids and their hosts and their co-evolution in biogeographic context are questions of outstanding interest. Here we report on our endeavour to gain insight into some of these questions by analysing five new plasmids from different *Thermococcus* strains that have been isolated from geographically remote hydrothermal vents.

Members of the order *Thermococcales* (genera *Thermococcus*, *Pyrococcus* and *Paleococcus*) are obligate heterotrophs that grow anaerobically at temperatures between 70 and 105°C. *Thermococcales* normally thrive in geothermal aquatic environments, mostly in deep-sea hydrothermal vents, where they play a major role in the ecology and metabolic activity of microbial consortia [1]. Plasmids are relatively common in *Thermococcales*; ∼40% of isolates (n>190) were found to contain extrachromosomal elements [2]–[4]. However, complete sequences are currently available for only eight of them (reviewed in [5]). Based on the type of replication protein they encode, plasmids from *Thermococcales* can be classified into five groups/families (Table S1 1). (i) Two related plasmids pGT5 (3,4 kb; *P. abyssi* GE5) and pTN1 (3,6 kb; *T. nautilus* 30/1), each encoding only two proteins, replicate using the rolling-circle mechanism [6]–[8]. (ii) Another small plasmid, pRT1 (3,4 kb; *Pyrococcus* strain JT1), also encodes two proteins [9], which, however, do not share sequence similarity with the proteins of pGT5/pTN1. The putative replication protein p63 of pRT1 is not closely related to other protein in the databases, except for the corresponding protein in pAMT11, a much bigger plasmid (20,5 kb) recently isolated from *Thermococcus* sp. AMT11 [10]. (iii) Plasmid pT26-2 (21,6 kb; *Thermococcus* sp. 26/2) shares a number of genes with putative integrated elements residing in the genomes of various *Thermococcales* and *Methanococcales* [11]. (iv) The largest of the thermococcal plasmids reported to date, pTBMP1 (54,2 kb) of *T. barophilus* MP [12], does not appear to be related to any of the plasmids listed above. (v) Finally, two related plasmids, pTN2 (13,0 kb) and pP12-1 (12,2 kb) have been isolated from *T. nautilus* 30/1 and *Pyrococcus* sp. 12/1, respectively [11].

Here we report on isolation and sequencing of five new thermococcal plasmids isolated from hydrothermal vents located in Atlantic, Pacific, and Indian Oceans. Based on gene content and phylogenetic analyses, the plasmids could be ascribed to two subfamilies, one of which includes the previously described plasmids pTN2 and pP12-1. Our analyses established a clear evolutionary link between thermococcal plasmids and the *Pyrococcus abyssi* virus 1 and also uncovered a recent horizontal plasmid transfer from *Thermococcales* to *Methanococcales*.

## Results and Discussion

### Preliminary characterisation of five novel plasmid-carrying *Thermococcus spp.*

**Figure 1:**
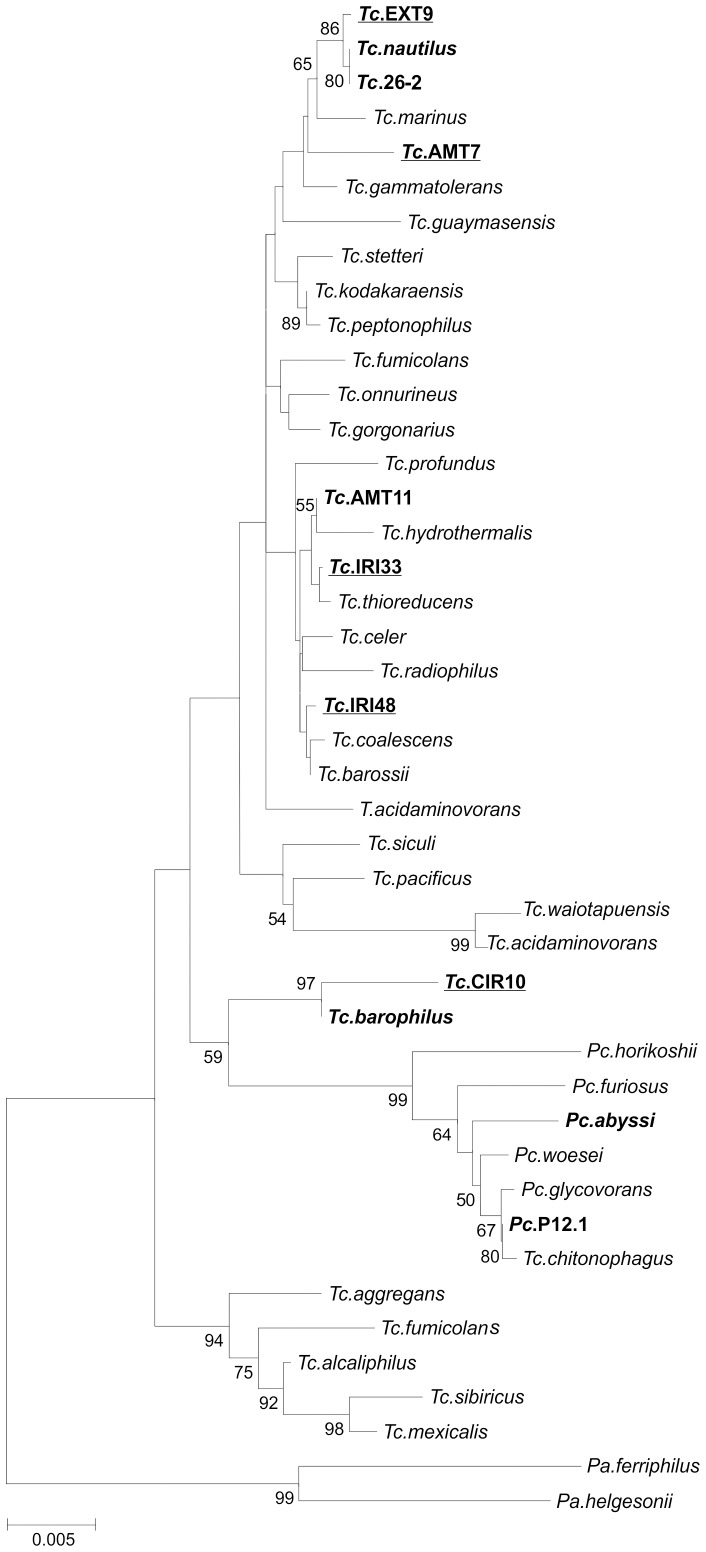
Phylogenetic tree of *Thermococcales* based on 16S rRNA gene sequences. The evolutionary history was inferred by using the Maximum Likelihood method based on the Tamura-Nei model. The bootstrap consensus tree inferred from 100 replicates. A discrete Gamma distribution was used to model evolutionary rate differences among sites (4 categories (+*G*, parameter = 0.1068)). The rate variation model allowed for some sites to be evolutionarily invariable ([+*I*], 75.0448% sites). The tree is drawn to scale, with branch lengths measured in the number of substitutions per site. The analysis involved 45 nucleotide sequences. All positions with less than 95% site coverage were eliminated. That is, fewer than 5% alignment gaps, missing data, and ambiguous bases were allowed at any position. There were a total of 1232 positions in the final dataset. Strains carrying sequenced plasmids are indicated by bold characters. The five plasmid-carrying *Thermococcus* strains isolated and characterized in this study are underlined.

The five *Thermococcus* strains carrying the plasmids described in this study were isolated from rock samples collected from black smoker chimneys located in distinct deep hydrothermal sites of three oceans (Atlantic, Pacific and Indian), at depths varying between 2274 and 2508 m (see Materials and Methods for details). They all grew between 60–90°C and pH 5–8.5 (with optima around 80–85°C and pH 6.5–7.0) under strictly anaerobic conditions. Assignment of the novel isolates to the *Thermococcus* genus was confirmed by phylogenetic analysis of their near-complete 16S rRNA genes (Fig. 1). Strains IRI33 and IRI48, although originating from the Mid-Atlantic Ridge, grouped together with *Thermococcus* sp. strain AMT11 harbouring a 20 kb plasmid [10] and *T. barossii* [13], both of which were isolated from black smokers of the East Pacific Rise. Strains AMT7 and EXT9 belong to a cluster containing two other plasmid-carrying isolates, strains 26-2 and 30-1, assigned to the *T. nautilus* species [8], [11]. The last isolate, CIR10, belongs to a separate clade with the closest related species being *T. barophilus*.

### General features of the novel plasmids of *Thermococcus spp.*

**Figure 2:**
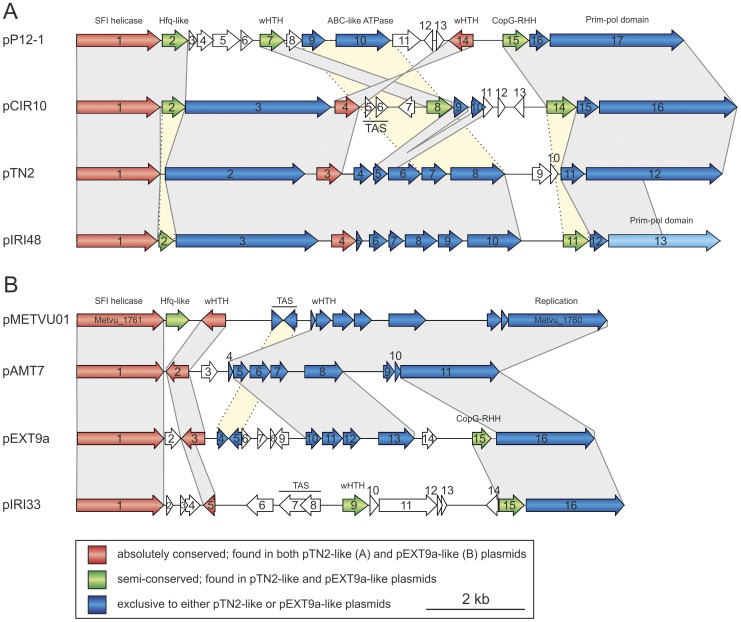
Comparative genomic analysis of the five novel thermococcal plasmids. (A) pTN2-like and (B) pEXT9a-like plasmids Genes absolutely conserved in all eight plasmids are shown in red; the semi-conserved genes present in some members of both plasmid groups are in green; conserved genes restricted to either one of the two groups are depicted in blue. Abbreviations: SFI, superfamily I; wHTH, winged helix-turn-helix motif; ABC, ATP-binding cassette; RHH, ribbon-helix-helix motif; Prim-pol, primase-polymerase; TAS, toxin-antitoxin system. General characteristics of the plasmids depicted in this Figure can be found in Table 1.

Plasmids pIRI33, pIR48, pAMT7, pEXT9 and pCIR10 were isolated from their respective host strains using a modified alkaline lysis method as previously described [10]. Southern hybridizations of total DNAs from each isolate with plasmid-specific probes revealed a relationship between the five novel plasmids (not shown) and the lack of putative integrated copies of these plasmids in their host chromosomes. The five novel plasmids were completely sequenced and their general characteristics are presented in Table 1. Analysis of their gene content revealed that the five plasmids form a single family, which also includes the previously sequenced *Thermococcus nautilus* plasmid pTN2, *Pyrococcus* sp. 12/1 plasmid pP12-1 [11] and, unexpectedly, *Methanocaldococcus vulcanius* M7 plasmid pMETVU01. Members within this family contain two invariable genes and an overlapping, semi-conserved set of genes (described below). Gene content analysis allowed further delineation of the plasmids into two subfamilies, pTN2-like and pEXT9a-like (Fig. 2; Fig. S1). Consistently with this grouping, the plasmid size and coding density for the two subfamilies was also found to differ considerably (Table 1).

**Table 1:** General characteristics of the plasmids analysed in this study.

Plasmid	Organism	Origin	Length, bp	Coding, %	CDS	G+C%	Accession
pTN2	*Thermococcus nautilus*	East Pacific Rise 13°N (−2330 m)	13015	91,1	12	48,4	NC_014115
pP12-1	*Pyrococcus* sp. 12/1	East Pacific Rise 13°N (−2330 m)	12205	90,8	17	44,7	NC_014110
pCIR10	*Thermococcus* sp.	Central Indian Ridge TJ (−2420 m)	13322	90,3	16	45,5	this study
pIRI48	*Thermococcus* sp.	Mid-Atlantic Ridge 36°N (−2274 m)	12974	90,3	13	50,0	this study
pMETVU01	*Methanocaldococcus vulcanius* M7	East Pacific Rise 13°N (−2600 m)	10704	72.2	13	46,3	NC_013408
pAMT7	*Thermococcus* sp.	East Pacific Rise 13°N (−2330 m)	8576	80,9	11	45,6	this study
pEXT9a	*Thermococcus* sp.	East Pacific Rise 9°N (−2508 m)	10556	80,6	16	45,8	this study
pIRI33	*Thermococcus* sp.	Mid-Atlantic Ridge 36°N (−2274 m)	11041	80,9	16	44,9	this study

### Plasmid gene content

#### Replication proteins

One of the two absolutely conserved genes present in both pTN2-like and pEXT9a-like plasmids encodes a superfamily I (SFI) helicase (Fig. 2), which has been also previously identified in pTN2 and pP12-1 [11]. Sequence analysis revealed that all conserved motifs characteristic to UvrD/REP-like helicases, except for the Q motif [14], are conserved in the plasmid homologues (data not shown). Although the exact role of UvrD/REP-like helicases *in vivo* is obscure, they are known to unwind the dsDNA duplex and promote replication of mobile genetic elements, such as viruses (e.g., phiX174, M13) and plasmids [15], [16]. It is therefore likely that SFI helicase homologues encoded by archaeal plasmids are responsible for unwinding of the dsDNA duplex during plasmid replication.

**Figure 3:**
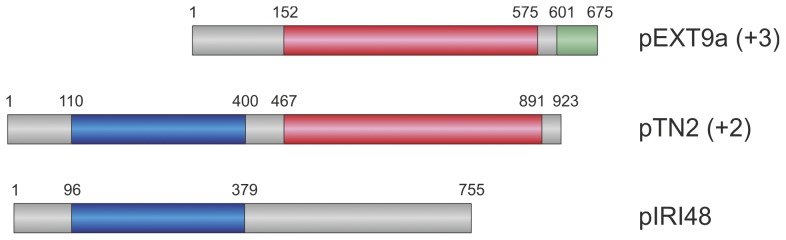
Modular relationship among plasmid-encoded replication proteins. Horizontal bars represent replication proteins with distinct domains indicated with different colours – the primase-polymerase domain is shown in blue, the C-terminal domain of pTN2 replicase is in red, and the winged helix-turn-helix domain is in green. The numbers above the bars represent the amino acid coordinates of the depicted domains in the replicases of respective plasmids (plasmid names are on the right). The numbers next to the plasmid names denote how many additional plasmids encode the specific type of a replication protein.

Immediately upstream of the helicase genes all plasmids possess large ORFs (>650 codons; Table S2). One of these, from pTN2 (gene *12* in Fig. 2A), has been demonstrated to encode a functional DNA primase-polymerase [11], suggesting that ORFs in other plasmids might also encode plasmid replication proteins. Notably, the protein sequences from different plasmids are not strictly homologous, but rather display a modular relationship (Fig. 3). Sequence analysis of the primase-polymerase from pTN2, protein tn2-12p, revealed that the protein is composed of at least two distinct domains: the N-terminal prim-pol domain, which shares similar features with members of the Archaeo-eukaryotic primase (AEP) superfamily [11], [17], and the C-terminal domain, which is not significantly similar to proteins in the extant databases, except for homologues in related plasmids. True homologues of the pTN2 primase-polymerase are encoded by plasmids pP12-1 and pCIR10 (Fig. 2A). Interestingly, pIRI48, which based on the gene content also belongs to the pTN2-like subfamily (Fig. 2A), encodes a different protein variant. Similarly to tn2-12p-like proteins, pIRI48 gp13 possesses an N-terminal prim-pol domain, but the C-terminal domains of these proteins are unrelated (Fig. 3). A yet different protein is encoded by all pEXT9a-like plasmids. The latter group is also related to tn2-12p-like proteins, but unlike in pIRI48 gp13, the relationship is confined to the C-terminal (∼28% identity over 458 aa region for pEXT9a gp16; Table S2) rather than the N-terminal domain (Fig. 3). Instead of the prim-pol domain, the pEXT9a-like proteins possess a ∼150 aa N-terminal domain of unknown function and provenance. Furthermore, unlike in the pTN2-like group, pEXT9a-like proteins possess a C-terminal extension that is predicted to adopt a DNA-binding winged helix-turn-helix (wHTH) fold (Table S2, Fig. 3). Although without experimental evidence it is not possible at the moment to predict the biochemical activity of pEXT9a-like proteins, the genetic neighbourhood as well as fusion to the prim-pol domain in pTN2-like plasmids strongly suggests that these proteins represent a new family of DNA replication proteins.

Genes encoding genome replication proteins are undoubtedly amongst the most important functional determinants of any replicon, be it a virus, a plasmid or a cellular chromosome. It is thus surprising with what ease and apparent frequency these genes are swopped between different mobile elements; numerous cases of non-homologous displacements have been reported both in bacteria and in archaea [10], [11], [18], [19], raising a question of whether replication protein genes should be considered as “core genes” when reconstructing the evolutionary history of a given group of mobile genetic elements, especially in the case of small replicons [10], [20]. The three different variants of putative replicases encoded by the thermococcal plasmids (Fig. 3) further illustrate this point.

**Figure 4:**
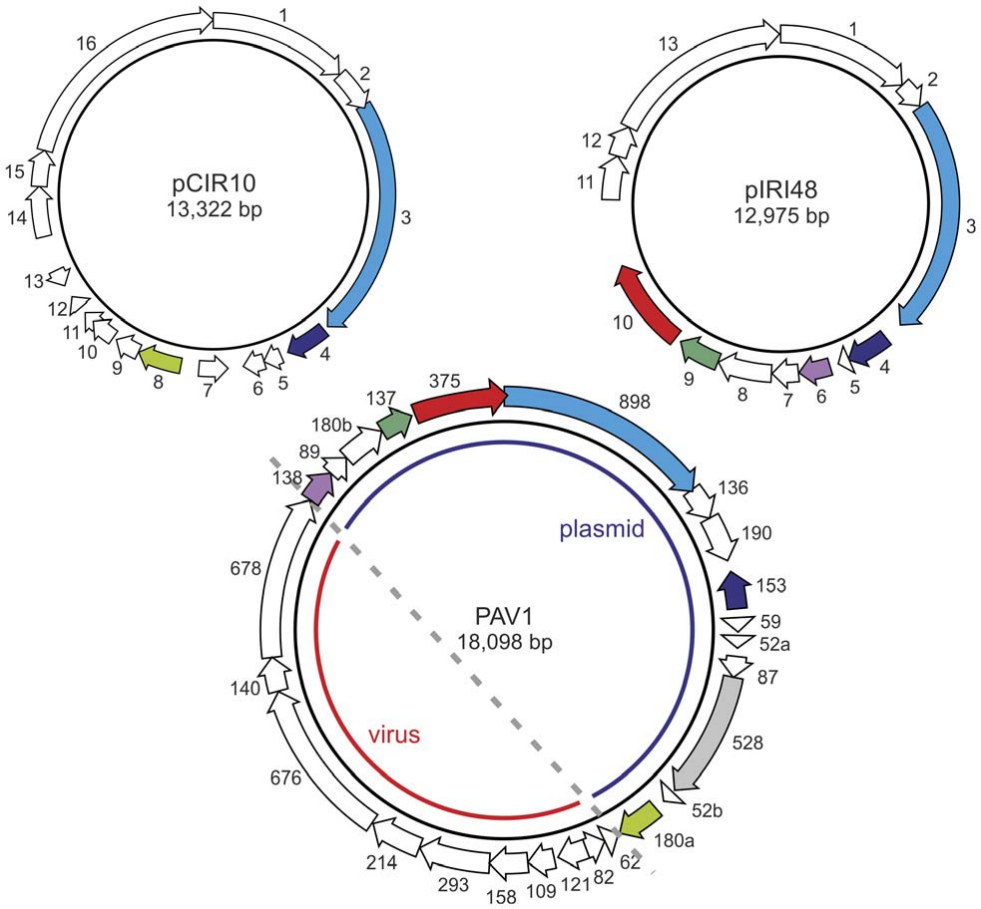
Phylogenetic analysis of the plasmid-encoded superfamily I helicases. The plasmid names are coloured according to the geographical origin of the strains from which they were isolated: blue, East Pacific Ocean ridge; red, Mid-Atlantic Ocean ridge; green, Indian Ocean triple junction. Replicase–helicase (*rep* and *hel*) gene cassettes are shown on the right; domain organizations of the replication proteins encoded by plasmids within the three phylogenetic clades (1–3) are indicated. The plasmid of *Methanocaldococcus vulcanius* M7 is boxed. The evolutionary history was inferred by using the Maximum Likelihood method based on the JTT model (+I, +G [4 categories]). Numbers at the branch-points represent bootstrap values (100 replicates). The scale bar represents the number of substitutions per site. The analysis involved 12 amino acid sequences. All positions containing gaps and missing data were eliminated. There were a total of 398 positions in the final dataset. The tree was rooted with helicases from haloarchaea, based on previous phylogenetic analysis [11]. GenBank accession numbers: *Halorubrum lacusprofundi* ATCC 49239, YP_002564878; *Halogeometricum borinquense* DSM 11551, YP_004037766; *Thermococcus onnurineus* NA1, YP_002307767; *Thermococcus gammatolerans* EJ3 integrating element TGV2, YP_002959996; pMETVU01, YP_003248093; pTN2, YP_003603582; pP12-1, YP_003603439.

To gain additional insights into the evolution of the thermococcal plasmid replication proteins, we performed a Maximum-likelihood analysis of the SFI helicase proteins conserved in both pTN2-like and pEXT9a-like plasmids. Previous phylogenetic analysis of the SFI helicases showed that pTN2 and pP12-1 helicases are most closely related to corresponding proteins from *Thermococcales* (*T. onnurineus* NA1 and *T. gammatolerans* EJ3) and *Halobacteriales*, together forming a monophyletic group, separate from other archaeal, bacterial and eukaryotic SFI helicases [11]. We therefore concentrated on thermococcal helicases and used halobacterial sequences as outgroups (Fig. 4). In our analysis, thermococcal helicases formed three separate clades. Interestingly, these clades coincided with the groups defined based on the type of the replication protein encoded by the plasmids (Fig. 3, 4). Clade 1 contains all pEXT9a-like plasmids that encode a putative replication protein displaying sequence similarity to the C-terminal domain of the pTN2 primase-polymerase (Fig. 4). Clade 2 contains plasmids pTN2, pP12-1 and pCIR10, all of which encode pTN2-like replication proteins. Interestingly, plasmid pIRI48, despite being the most similar one to pTN2 based on the common gene content (Table S2), falls into clade 3 together with *T. onnurineus* NA1 and *T. gammatolerans* EJ3. As described above, the replication protein of pIRI48 shares the N-terminal prim-pol domain with the pTN2 protein, but contains an unrelated C-terminal domain (Fig. 3). Notably, the SFI helicase gene in *T. gammatolerans* EJ3 genome resides within the IE, previously designated as TGV2 [21], and is preceded by a gene encoding a putative replication protein containing an N-terminal prim-pol domain (best hit to the corresponding domain of pIRI48 gp13; 29% identity over 201 aa region) and a unique C-terminal domain, not related to any of those found in replicases of either pTN2-like or pEXT9a-like plasmids. In *T. onnurineus* NA1 genome the helicase (TON_1380) gene is preceded by primase-polymerase- (TON_1379) and integrase-encoding (TON_1378) genes, suggesting that this three-gene cassette might have also arisen from an integration of a mobile element. Interestingly, the putative primase-polymerase encoded within the *T. onnurineus* NA1 genome represents a yet another variant of this group of proteins; the protein possesses an N-terminal prim-pol domain and a unique C-terminal domain. Thus, all three replicases encoded by elements belonging to the helicase-based clade 3 (Fig. 4) possess related N-terminal, but distinct C-terminal domains. The fact that the helicase sequences formed phylogenetic clades coinciding with the grouping defined on the basis of the replication protein types rather than the overall gene content similarity of respective plasmids (Table S2) points to the co-evolution of these helicases with their cognate replicases. It is possible that the co-evolution is dictated by the necessity to preserve protein-protein interaction between the corresponding proteins during plasmid DNA replication.

#### Origins of replication

In both pTN2-like and pEXT9a-like plasmids the operon encoding the two replication proteins is preceded by large intergenic regions (Fig. 2). Based on the cumulative GC skew analysis and presence of repetitive sequences, these regions were predicted to contain the origin of replication (*ori*) in pTN2 and pP12-1 [11]. In an attempt to identify a possible *ori* site for the plasmids in our dataset, we performed a cumulative GC skew analysis. While the intergenic region preceding the replication gene cassette was found to be consistent with the location of the *ori* site in pIRI48, pAMT7, pEXT9a and pMETVU01 (detection of minima in GC skew and presence of the most significant sequence repeats in this region), the situation is more difficult to interpret for plasmids pCIR10 and pIRI33 (Fig. S2). For example, GC skew graph of pIRI33 shows two valleys, one of which coincides with the typical predicted *ori* location for other related plasmids, while the second, deeper one, is in the intergenic region between genes *8* and *9*. This could either suggests that the *ori* site in pIRI33 has shifted to this new position, possibly after horizontal acquisition of a new *ori* from an unrelated replicon along with the genes for the toxin-antitoxin genes (see below) or that this gene transfer event has perturbed the GC skew, rendering the prediction of *ori* site more complicated.

#### DNA-binding proteins

The second of the two genes absolutely conserved in both pTN2-like and pEXT9a-like plasmids (Fig. 2; gene *3* in pTN2 and pEXT9a) potentially encodes a ∼170 aa protein. This group of proteins contains an N-terminal coiled-coil domain followed by a predicted wHTH domain (Suplementary Files 4 and 6), suggesting an involvement in transcription regulation. Two additional groups of putative wHTH transcription factors are encoded by some of the pTN2-like and pEXT9a-like plasmids (Fig. 2).

Another group of putative DNA-binding proteins that is semi-conserved in the pTN2-like (pP12-1, gene *15*; pCIR10, gene *14*; pIRI48, gene *11*) and pEXT9a-like (pEXT9a, gene *15*; pIRI33, gene *15*) plasmids consists of proteins containing an N-terminal ribbon-helix-helix (RHH) motif (Fig. S4). RHH proteins in most cases function as dimers and can either negatively or positively regulate the expression of the target genes. In the *Sulfolobus* plasmid pRN1 (and other pRN-like plasmids), an RHH protein Orf56 is encoded upstream of the primase-polymerase gene (*orf904*) and negatively regulates the expression of the replicase by repressing the transcription of the *orf56*-*orf904* co-transcript [22]. The proximity of RHH protein-coding genes to the replication gene cassettes in thermococcal plasmids suggests a similar regulatory role for these putative transcription factors.

#### RNA-binding Sm/Hfq-like proteins

Another peculiar group of proteins potentially involved in nucleic acid-binding is encoded by plasmids pP12-1 (gene *2*), pCIR10 (gene *2*), pIRI48 (gene *2*) and pMETVU01 (gene Metvu_1762). These proteins bear a predicted C-terminal Hfq-like domain and, with the exception of pIRI48 gp2, an N-terminal C2H2 Zn-finger (ZF) domain (Table S2 and Fig. S5). Notably, in all plasmids the genes for these proteins are situated immediately downstream of the helicase genes and appear to belong to the same transcriptional unit. Hfq-like proteins belong to a family of Sm proteins that perform a range of important RNA-related functions in cellular organisms belonging to all three domains of life [23].

The fusion of the ZF domain to the Hfq-like domain is, to our knowledge, unique to the plasmid-encoded proteins described here. The role of the ZF domain in these proteins is not clear, however. It is possible that the ZF recruits the protein to the DNA for targeted regulation of certain transcripts. Indeed, it has been recently demonstrated that *E. coli* Hfq modulates transcription, presumably by binding to nascent transcripts [24]. It has also been shown that Hfq is one of the three major nucleoid proteins during the exponential growth phase and preferentially, although weakly, binds to curved DNA [25]. Due to the ability to bind both RNA and DNA, it was suggested that Hfq might be involved in coupling transcription to translation. It is tempting to speculate that the plasmid-encoded Hfq-like proteins perform a similar role during plasmids' replication.

PSI-BLAST analysis revealed that proteins homologous to pCIR10 gp2 (containing both ZF and Hfq-like domains) are also encoded by three unrelated methanococcal plasmids, namely ECE1 (NP_044153) and ECE2 (NP_044176) of *M. jannaschii* DSM 2661 as well as pFS01 (YP_003459249) of *Methanocaldococcus* sp. FS406-22. Furthermore, recent structural studies revealed a variant of an Hfq-like protein encoded by the *Pyrobaculum* spherical virus [26], suggesting that the role of Hfq-like proteins in the replication of mobile elements in (hyperthermophilic) archaea might be more significant than currently appreciated.

#### Toxin-antitoxin genes

The thermococcal plasmids carry genes for two distinct toxin-antitoxin (TA) systems. The putative TA genes of the RelBE family are encoded by plasmids pCIR10 (genes *5/6*), pEXT9a (genes *4/5*) and pMETVU01 (genes Metvu_1751/Metvu_1752) (Fig. S6), while pIRI33 (genes *7/8*) encodes a TA of the VapBC (also known as RHH/PIN) family [27], [28].

In the RelBE TA system, RelE is a ribonuclease, which inhibits translation during nutritional stress by cleaving mRNAs positioned at ribosomal A-sites, whereas RelB antagonizes the action of RelE by direct protein–protein interaction and repression of *relBE* operon transcription [27]. The homologues of the plasmid-encoded RelBE proteins are widespread in bacteria and archaea, with the closest homologues being encoded in the genomes of *Thermococcales* (Table S2). Interestingly, phylogenetic analysis of RelE-like proteins encoded by the three plasmids (pCIR10, pEXT9a and pMETVU01) as well as several selected archaeal genomes (Fig. S7) suggests that RelBE loci of pEXT9a and pMETVU01 are orthologous, while the one in pCIR10 has been acquired independently from a distinct source.

VapBC TA are abundant in bacteria and even more so in archaea [27]. In enterobacteria, VapC toxins are PIN (**Pi**lT **N**-terminal) domain-containing site-specific endonucleases that cleave tRNA(fMet) in the anticodon stem-loop thereby inhibiting protein translation [29]. The antitoxin VapB proteins counteract the toxic action of VapCs by direct protein-protein interaction. Unlike VapCs, VapBs do not form a homogeneous protein group and typically possess DNA-binding domains of at least four different classes, including RHH, HTH, PHD/YefM and AbrB [27], [28]. Gene *7* of pIRI33 encodes a PIN domain (COG1569) protein with numerous homologues in bacterial and archaeal genomes (Table S2). The gene, located immediately upstream of the *vapC* toxin gene and overlapping the latter by 119 nt (Fig. 2B), encodes a putative RHH protein (94% HHpred probability; Table S2), which appears to represent an antitoxin component of the VapBC system.

TA loci are abundantly found in bacterial and archaeal genomes as well as in bacterial plasmids and a few bacterioviruses [27], [28]. We have also observed TA genes to be present within proviruses [30] and integrative elements [31] of *Methanococcales*. However, to the best of our knowledge, TA genes have not been previously reported in any of the archaeal extrachromosomal elements, including viruses and plasmids. In bacterial mobile elements TA loci have been experimentally shown to contribute to the stable maintenance of these replicons within their host cells [32]. A similar function is therefore most likely for TA encoded by archaeal plasmids.

### Relationship to *Pyrococcus abyssi* virus 1

**Figure 5:**
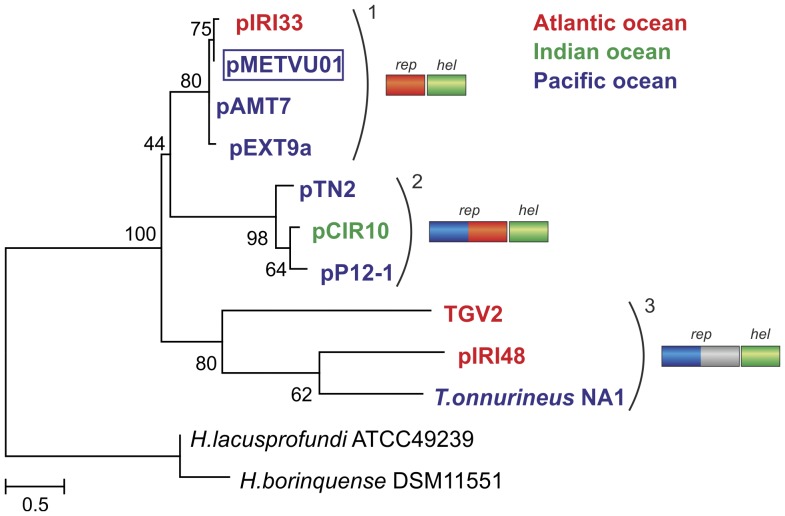
Relationship between thermococcal plasmids and virus PAV1. Homologous genes are coloured similarly. PAV1 ORF528, which has homologues in haloarchaeal plasmids, is shaded grey.

Analysis of thermococcal plasmids pTN2 and pP12-1 has previously revealed that these plasmids share three common genes with the *Pyrococcus abyssi* virus 1 (PAV1) [5], [11]. PAV1 virions display a spindle-shaped morphology and contain a circular dsDNA genome of 18 kb [33], [34]. Our analysis confirms and further extends the previous observation of genetic relatedness between thermococcal plasmids and PAV1. Comparative genomic analysis revealed six PAV1 genes that have homologues in thermococcal plasmids (Fig. 5; Fig. S3). One of these genes (PAV1 ORF153) is absolutely conserved in all pTN2-like and pEXT9a-like plasmids and encodes the putative transcriptional regulator with an N-terminal coiled-coil domain and a C-terminal wHTH domain (see above). PAV1 ORF180a has homologues in plasmids pIRI33 (gene *9*), pCIR10 (gene *8*) and pP12-1 (gene *7*) and also encodes a putative wHTH domain containing transcriptional regulator. Homologues of gene ORF138 are present in plasmids pIRI48 (gene *6*) and pTN2 (gene *4*), while the largest of the PAV1 genes, ORF898, has homologues in plasmids pCIR10 (gene *3*), pTN2 (gene *2*) and pIRI48 (gene *3*). Proteins from the latter group all possess a predicted coiled-coil region, but do not display appreciable sequence similarity to proteins in the public databases. Homologues of genes ORF137 and ORF375 are found in tandem organization and are present in thermococcal plasmids pP12-1 (genes *9* and *10*), pIRI48 (genes *9* and *10*) and pTN2 (genes *7* and *8*). The two genes along with gene ORF180b form a three-gene cassette, which is also conserved within the putative provirus A3 VLP of *Methanococcus voltae* A3 (genes Mvol_0500/Mvol_0499/Mvol_0498) [35]. ORF137 homologues encode proteins of unknown functions, while those of ORF375 encode P-loop ATPases, with readily discernible Walker A and B motifs. PSI-BLAST and HHpred analyses point to the relatedness of ORF375-like proteins to ABC transporters (Table S2). However, the function of these proteins in the propagation of the mobile elements remains obscure. Thorough analysis of the PAV1 genome did not reveal any other genes that would have counterparts in the currently available thermococcal plasmids. Notably, however, ORF528, which encodes a putative wHTH protein [33], displays significant sequence similarity with proteins from several haloarchaeal plasmids, namely *Halalkalicoccus jeotgali* B3 plasmid 2 (YP_003738738 and YP_327788; 24% identity over 328 aa region), *Natronomonas pharaonis* DSM 2160 plasmid PL23 (YP_327788; 25% identity over 163 aa region) and *Haloquadratum walsbyi* C23 plasmid PL6A (CCC41961; 24% identity over 187 aa region).

Interestingly, all the genes that have homologues in archaeal plasmids and integrating elements are clustered together and occupy roughly half of the PAV1 genome (Fig. 5), while genes that were shown (ORF121) or predicted (ORF676, ORF678) to encode structural virion proteins are located in the other half [33]. Notably, ORF676 and ORF678 are the only two ORFs which are shared between PAV1 and another recently isolated thermococcal spindle-shaped virus, *Thermococcus prieurii* virus 1 [36]. It is therefore tempting to speculate that PAV1-like viruses emerged as a result of recombination between two distinct types of mobile genetic elements (MGEs), a plasmid and a virus, which respectively donated genetic determinants for genome propagation and virion formation. Alternatively, loss of the genetic determinants for virion structure from the PAV1-like viral genome might have given rise to the pTN2-like family of plasmids. Indeed, genetic fusions between plasmids and viral genomes have been suggested to play a central role in the emergence and evolution of certain bacterial and archaeal viruses [37], [38]. Intimate interplay between viruses and plasmids has been previously observed in Archaea. For example, upon superinfection with spindle-shaped fuselloviruses, crenarchaeal pRN-like plasmids pSSVi and pSSVx are encapsidated into virus-like particles that are released from the cells and propagate in a virus-like fashion [39], [40]. The extent of genomic relationship between PAV1 and pTN2-like plasmids suggests that there might also be functional interaction between these two types of replicons and testing the latter possibility might prove to be highly rewarding. Additional genome sequences of PAV1-like viruses are required to obtain a more comprehensive picture of the genomic relationship between these archaeal viruses and plasmids.

### Horizontal plasmid transfer: from *Thermococcales* to *Methanococcales*

Previous studies have revealed close genetic relationship between plasmids and integrating elements (IEs) of *Thermococcales* and *Methanococcales* [5], [11], [31]. More specifically, members of *Thermococcales* and *Methanococcales* were found to share a group of MGEs related to thermococcal plasmid pT26-2 [11]. Phylogenetic analysis of six concatenated core proteins conserved in all these MGEs revealed a clear separation of the elements belonging to the two archaeal orders, with the phylogenetic tree being roughly congruent with the species tree of *Archaea*. Consequently, it was suggested that pT26-2 and related IEs have co-evolved with their hosts and diverged from an ancestor that already propagated in *Archaea* before the divergence between *Methanococcales* and *Thermococcales*. High level of similarity in gene content between the thermococcal pEXT9a-like plasmids and the methanococcal pMETVU01 prompted us to test the latter hypothesis in the case of plasmids studied here.

In our phylogenetic reconstruction of the plasmid-encoded helicase proteins (Fig. 4), the sequence of methanococcal pMETVU01 helicase is robustly positioned within clade 1 along with the three pEXT9a-like plasmids. This position is also consistent with the comparative genomic analysis, which revealed that pMETVU01 is not related to any other methanococcal MGE. On the contrary, all thirteen putative genes of pMETVU01 have homologues within the pan-genome of pTN2-like and pEXT9a-like thermococcal plasmids (Fig. 2B). The highest number of genes is shared between pMETVU01 and pAMT7 (10 common genes; 53–97% identity at the protein level). This is also reflected in the gene content tree, where pMETVU01 and pAMT7 cluster together (Fig. S1). In 16S rRNA gene phylogeny *Methanocaldococcus vulcanius* M7 is robustly positioned within the clade including members of *Methanococcales* (Fig. S8), validating the assignment of this organism to the family *Methanocaldococcaceae* within the order *Methanococcales* [41]. Thus, given the fact that *M. vulcanius* M7—the host of pMETVU01 plasmid—is a hyperthermophile isolated from the same hydrothermal field as *Thermococcus sp.* strain AMT7 (the two sampling sites are only ∼7 km apart) [41], it is reasonable to conclude that the origin of pMETVU01-like plasmids in *Methanococcales* is a result of a relatively recent horizontal transfer from *Thermococcales*. It therefore appears that, unlike pT26-2-like plasmids that to co-evolve with their hosts [11], pEXT9a-like plasmids are more prone to horizontal transfer. This is the first observation of a clear-cut horizontal plasmid transfer (HPT) between the organisms belonging to two different orders of *Euryarchaeota*. Importantly, such HPT might also play an important role in shuttling cellular genes between different organisms in hyperthermophilic environments.

An outstanding question is how such transfer is achieved. Neither pTN2-like nor pEXT9a-like plasmids display signatures of canonical conjugative plasmids [42], [43] and therefore are unlikely to spread by this mechanism. Notably, certain members of *Methanococcales* and *Thermococcales* have been shown to be naturally competent, capable of exogenous DNA uptake [44], [45]. This suggests that plasmid transfer between the organisms of two groups of hyperthermophilic archaea might occur by natural transformation. As mentioned above, *Sulfolobus* fuselloviruses mediate transfer of pRN-like plasmids pSSVi and pSSVx by encapsidating them into virus-like particles [39], [40]. Given the extent of similarity between PAV1 and thermococcal plasmids (Fig. 5), the possibility of a virus-assisted plasmid transfer also appears to be a viable option. Recently, different *Thermococcus* strains were shown to produce membrane vesicles (MVs), which were found to be associated with chromosomal as well as plasmid DNA [5], [46], [47]. Consequently, MVs might serve as vehicles in horizontal plasmid transfer in hyperthermophilic environments. Future research should reveal which of the above mentioned (not mutually exclusive) mechanisms are accountable for plasmid shuttling between different archaeal cells.

Finally, plasmids sequenced during this study might be useful in developing novel shuttle vectors. From this perspective, the pEXT9a-like plasmids are of special interest due to their ability to replicate in members of both *Thermococcales* (pAMT7, pEXT9a and pIRI33) and *Methanococcales* (pMETVU01). It is now important to test whether the same plasmid can replicate in cell from both archaea orders. Furthermore, the TA genes carried by pEXT9a-like plasmids might prove to be useful for stable maintenance of the shuttle vectors.

## Materials and Methods

### Origin and cultivation of the new *Thermococcus* strains

The new *Thermococcus* strains described in this study were isolated and cultivated following previously established protocols [3], [48]. Detailed procedures can be found in the Materials and Methods S1.

### Plasmids isolation and sequencing

Plasmids were obtained from 50 ml cultures in late exponential phase (approximately 10^8^ cells/ml) of *Thermococcus* strains AMT7, EXT9, IRI33, IRI48 and CIR10 using a modified alkaline lysis method as previously described [8]. Shotgun plasmid libraries of clones of each *Thermococcus* plasmid were constructed in pUC18 vector and sequenced from both ends as described previously [10]. The complete plasmid sequences were deposited to the GenBank under the following accession numbers: JQ661332 (pAMT7), JQ661331 (pEXT9a), JQ661329 (pIRI33), JQ661328 (pIRI48) and JQ661330 (pCIR10).

### ssu rDNA sequencing

The near full length genes for 16S rRNA were amplified from genomic DNA of *Thermococcus sp.* strains IRI33, IR48, CIR10, AMT7 and EXT9 using the polymerase chain reaction (PCR) with primers Arc-8F (5′ TCC GGT TGA TCC TGC CRG 3′) and Universal 1492R (5′ GGT TAC CTTACG ACT T 3′) as described previously [10]. PCR products were cloned into pGEM-T Easy vector (Promega) with E. coli XL-Gold (Stratagene) competent cells as recipient. In each case three positive clones were sequenced using universal sequencing primers M13 forward and M13 reverse by Sanger method. Sequences were compared to other Thermococcales 16S rRNA gene sequences using the web interface of the Ribosomal Database Project release 10 [49].

### Sequence analysis

The ORFs were predicted with the following criteria: minimal length – 39 codons; start codons – ATG, GTG or TTG; stop codons – TAA, TAG or TGA. Precise localization of potential CDS was manually adjusted based on the location of consensus Shine-Dalgarno sequences. Predicted protein sequences of thermococcal plasmids were analysed using BLASTP and PSI-BLAST [50] searches against nonredundant protein database at NCBI. For distant homology detection, HHpred [51] and FFAS03 [52] were used. Annotation tables for all plasmids described in this study can be found in Supporting Information (Table S2). PAV1 homologues in thermococcal plasmids were identified by comparing all PAV1 proteins sequences against the local database containing protein sequences of thermococcal plasmids. The results can be found in Supporting Information (Table S3).

For phylogenetic analysis multiple sequence alignments were constructed using PROMALS3D [53] and MUSCLE [54], manually examined and edited if required. Sequence alignments were visualized using Jalview [55]. Maximum likelihood analysis was carried out using MEGA5 software [56].

For gene content trees, a binary (presence/absence) matrix of plasmid genes was constructed and used to calculate gene content distances with GeneContent program [57].

## Supporting Information

Figure S1 **Plasmid gene content tree.** The tree was constructed using GeneContent program (Gu et al. Bioinformatics, 2005; 21:1713–1714) and rooted with the pTBMP1 plasmid of *T. barophilus* MP, which shares with the rest of the plasmids a single gene. Plasmid names are coloured according to the geographical origin of the *Thermococcales* strains from which they were isolated: blue, East Pacific Ocean ridge; red, Mid-Atlantic Ocean ridge; green, Indian Ocean triple junction.
(TIF)

Figure S2 **Cumulative GC skew analysis of pTN2-like (A) and pEXT9a-like (B) plasmids.** The ORF maps for each of the plasmids are shown above the corresponding GC skew plots. The large intergenic regions housing the origins of plasmid replication (*ori*) are matched with the inflections in the GC skew plots by grey shading. The putative new *ori* site in pIRI33 is indicated with a red circle.
(TIF)

Figure S3 **Alignment of winged helix-turn-helix (wHTH) proteins conserved in pTN2-like and pEXT9a-like plasmids.** The alignment is coloured according to sequence conservation (% identity). The secondary structure determined from the X-ray structure of wHTH transcriptional repressor BigR from *Xylella fastidiosa* (PDB ID: 3PQK) is shown above the alignment with α helices and β strands represented by red rectangles and blue arrows, respectively. The predicted coiled-coil domain is represented with a green bar.
(TIF)

Figure S4 **Alignment of ribbon-helix-helix (RHH) motif containing proteins encoded by thermococcal plasmids.** The alignment is coloured according to sequence conservation (% identity). The secondary structure determined from the X-ray structure of RHH transcriptional repressor NikR from *Escherichia coli* (PDB ID: 2HZA) is shown above the alignment with α helices and β strands represented by red rectangles and blue arrows, respectively. The X-ray structure of the NikR dimer is shown on the right, with secondary structure elements coloured using the same code as depicted above the alignment.
(TIF)

Figure S5 **Alignment of Hfq-like proteins encoded by thermococcal plasmids.** The alignment is coloured according to sequence conservation (% identity). The N-terminal Zinc finger (ZF) domain of the plasmid proteins is aligned with the ZF domain of the human zinc-fingers and homeoboxes 1 protein (PDB ID: 2GHF), while the C-terminal domain is aligned with the Hfq-like proteins from *Methanocaldococcus jannaschii* (PDB ID: 2QTX) and *Staphylococcus aureus* (PDB ID: 1KQ1). The secondary structure elements displayed above the alignment (α helices – red rectangles, β strands – blue arrows) have been calculated from the respective X-ray structures. The X-ray structure of the hexameric Hfq-like protein from *Methanocaldococcus jannaschii* is shown on the right, with secondary structure elements coloured using the same code as depicted above the alignment.
(TIF)

Figure S6 **RelEB toxin-antitoxin system encoded by archaeal plasmids.** Plasmid-encoded RelE-like toxin (A) and RelB-like antitoxin (B) proteins are aligned with the corresponding protein from *Pyrococcus horikoshii* OT3 (PDB ID: 1WMI_A and 1WMI_B, respectively). The alignments are coloured according to sequence conservation (% identity). The secondary structure determined from the X-ray structures of corresponding *P. horikoshii* OT3 proteins is shown above the alignment with α helices and β strands represented by red rectangles and blue arrows, respectively. (C) The X-ray structure of the RelEB complex, with RelB and RelE shown in red and blue, respectively.
(TIF)

Figure S7 **Phylogenetic analysis of the cellular and plasmid-encoded RelE-like proteins.** The evolutionary history was inferred by using the Maximum Likelihood method based on the JTT model (+I, +G [4 categories]). The bootstrap consensus tree was inferred from 100 replicates. Numbers at the branch-points represent bootstrap values. Branches corresponding to partitions reproduced in less than 50% bootstrap replicates are collapsed. The scale bar represents the number of substitutions per site. The analysis involved 12 amino acid sequences. All positions containing gaps and missing data were eliminated. There were a total of 83 positions in the final dataset. Cellular RelE-like proteins are indicated by their GenBank identifiers followed by the organisms' names.
(TIF)

Figure S8 **Phylogenetic tree of selected archaeal species based on 16S rRNA gene sequences.** The evolutionary history was inferred by using the Maximum Likelihood method based on the Tamura-Nei model (+I, +G [4 categories]). The bootstrap consensus tree was inferred from 100 replicates. Numbers at the branch-points represent bootstrap values. Branches corresponding to partitions reproduced in less than 50% bootstrap replicates are collapsed. The scale bar represents the number of substitutions per site. The tree was rooted by using sequences of organisms belonging to the archaeal order *Methanobacteriales* as an outgroup. *Methanocaldococcus vulcanius* M7 is highlighted in red. The analysis involved 32 nucleotide sequences. All positions containing gaps and missing data were eliminated. There were a total of 1183 positions in the final dataset.
(TIF)

Materials and Methods S1 **Supporting Materials and Methods.**
(DOC)

Table S1 **Replication protein-based classification of** ***Thermococcales*** **plasmids.**
(DOC)

Table S2 **Annotation of the five plasmids described in this study.**
(DOC)

Table S3 **PAV1 ORFs with homologues in thermococcal plasmids.**
(DOC)
