# Supplementary figures and images for: Insights into Dynamics of Mobile Genetic Elements in Hyperthermophilic Environments from Five New Thermococcus Plasmids
Source: PLoS One. 2013 Jan 11;8(1):e49044. doi: 10.1371/journal.pone.0049044 (PMC3543421; doi:10.1371/journal.pone.0049044)

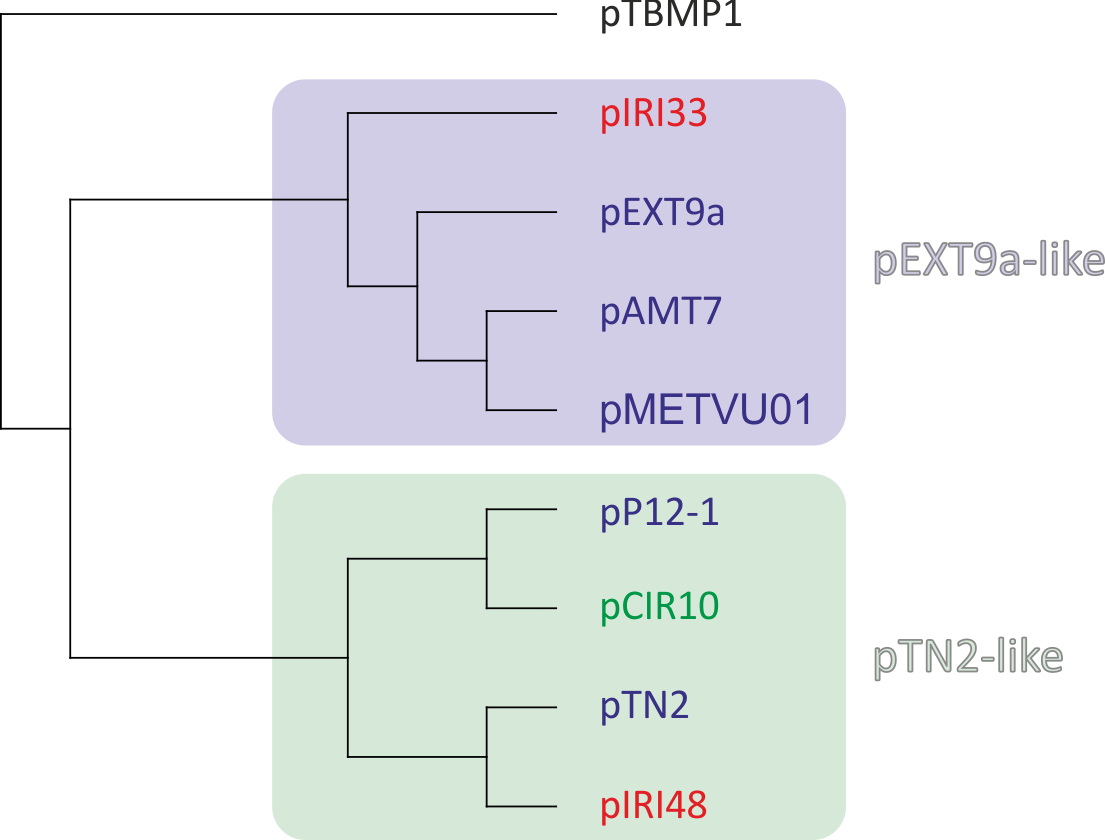

Supplement: Figure S1 — Plasmid gene content tree. The tree was constructed using GeneContent program (Gu et al. Bioinformatics, 2005; 21:1713–1714) and rooted with the pTBMP1 plasmid of T. barophilus MP, which shares with the rest of the plasmids a single gene. Plasmid names are coloured according to the geographical origin of the Thermococcales strains from which they were isolated: blue, East Pacific Ocean ridge; red, Mid-Atlantic Ocean ridge; green, Indian Ocean triple junction. (TIF) [file pone.0049044.s001.tif]

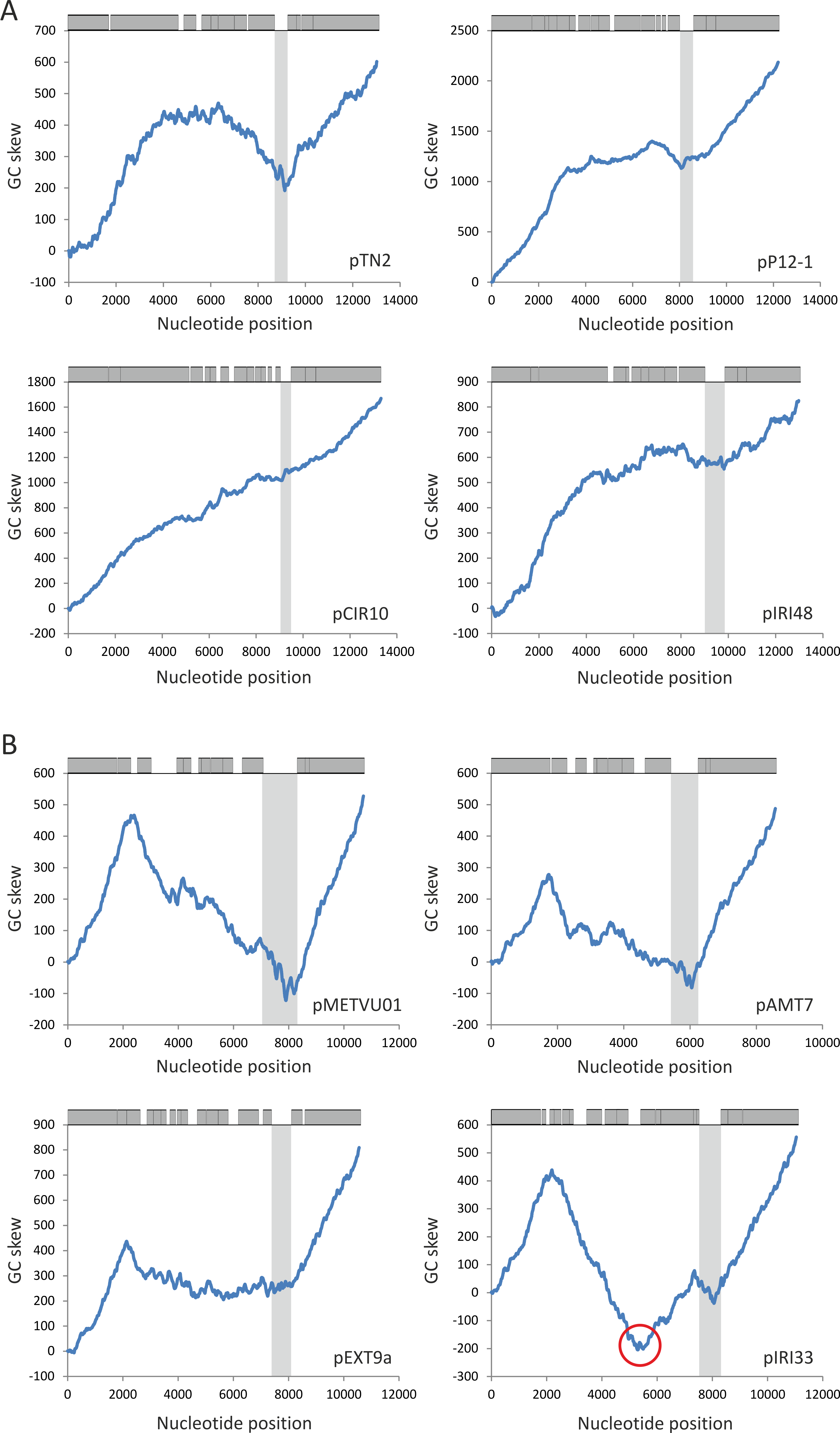

Supplement: Figure S2 — Cumulative GC skew analysis of pTN2-like (A) and pEXT9a-like (B) plasmids. The ORF maps for each of the plasmids are shown above the corresponding GC skew plots. The large intergenic regions housing the origins of plasmid replication (ori) are matched with the inflections in the GC skew plots by grey shading. The putative new ori site in pIRI33 is indicated with a red circle. (TIF) [file pone.0049044.s002.tif]

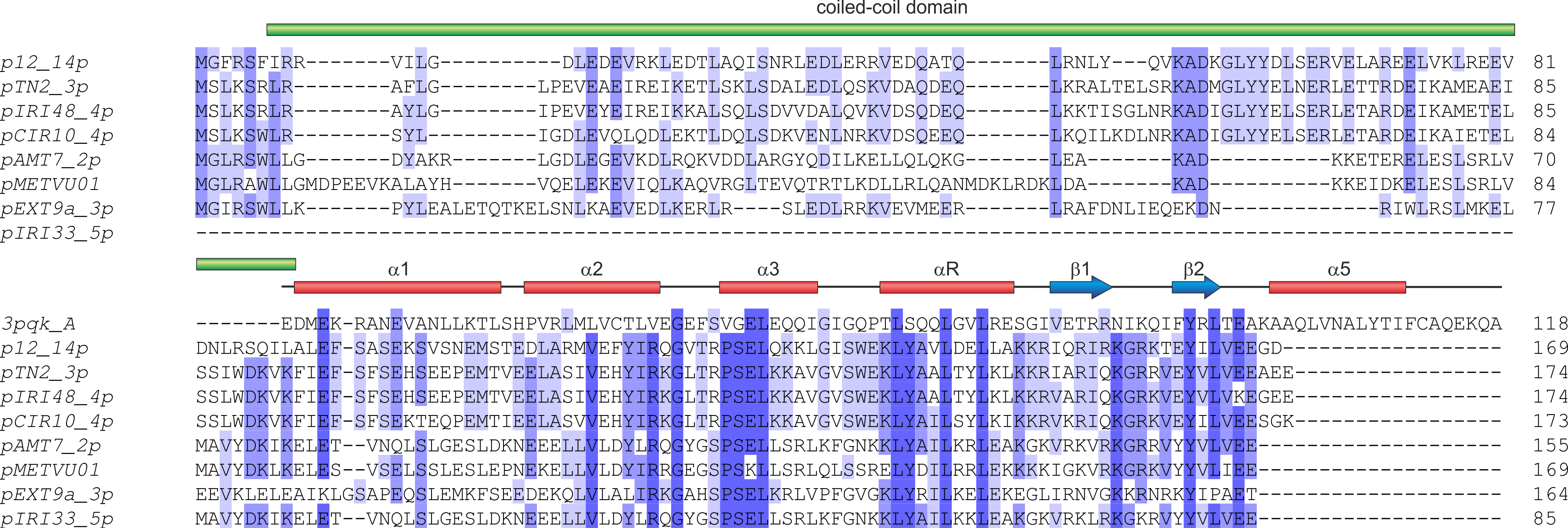

Supplement: Figure S3 — Alignment of winged helix-turn-helix (wHTH) proteins conserved in pTN2-like and pEXT9a-like plasmids. The alignment is coloured according to sequence conservation (% identity). The secondary structure determined from the X-ray structure of wHTH transcriptional repressor BigR from Xylella fastidiosa (PDB ID: 3PQK) is shown above the alignment with α helices and β strands represented by red rectangles and blue arrows, respectively. The predicted coiled-coil domain is represented with a green bar. (TIF) [file pone.0049044.s003.tif]

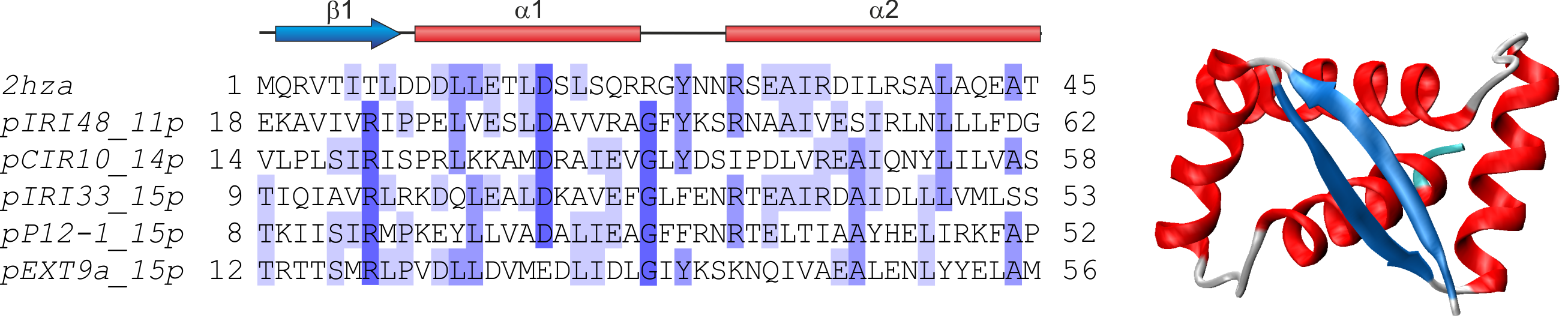

Supplement: Figure S4 — Alignment of ribbon-helix-helix (RHH) motif containing proteins encoded by thermococcal plasmids. The alignment is coloured according to sequence conservation (% identity). The secondary structure determined from the X-ray structure of RHH transcriptional repressor NikR from Escherichia coli (PDB ID: 2HZA) is shown above the alignment with α helices and β strands represented by red rectangles and blue arrows, respectively. The X-ray structure of the NikR dimer is shown on the right, with secondary structure elements coloured using the same code as depicted above the alignment. (TIF) [file pone.0049044.s004.tif]

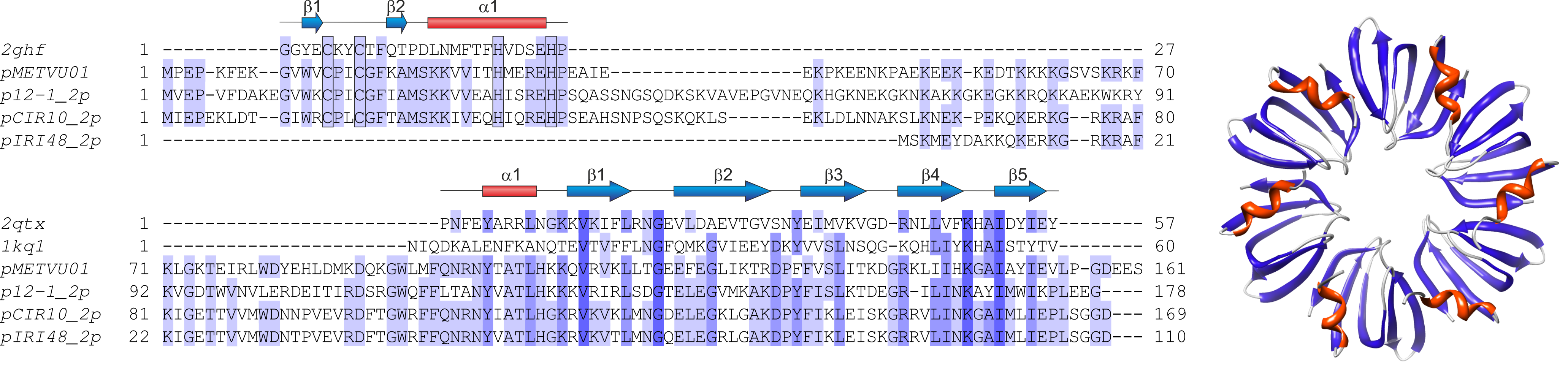

Supplement: Figure S5 — Alignment of Hfq-like proteins encoded by thermococcal plasmids. The alignment is coloured according to sequence conservation (% identity). The N-terminal Zinc finger (ZF) domain of the plasmid proteins is aligned with the ZF domain of the human zinc-fingers and homeoboxes 1 protein (PDB ID: 2GHF), while the C-terminal domain is aligned with the Hfq-like proteins from Methanocaldococcus jannaschii (PDB ID: 2QTX) and Staphylococcus aureus (PDB ID: 1KQ1). The secondary structure elements displayed above the alignment (α helices – red rectangles, β strands – blue arrows) have been calculated from the respective X-ray structures. The X-ray structure of the hexameric Hfq-like protein from Methanocaldococcus jannaschii is shown on the right, with secondary structure elements coloured using the same code as depicted above the alignment. (TIF) [file pone.0049044.s005.tif]

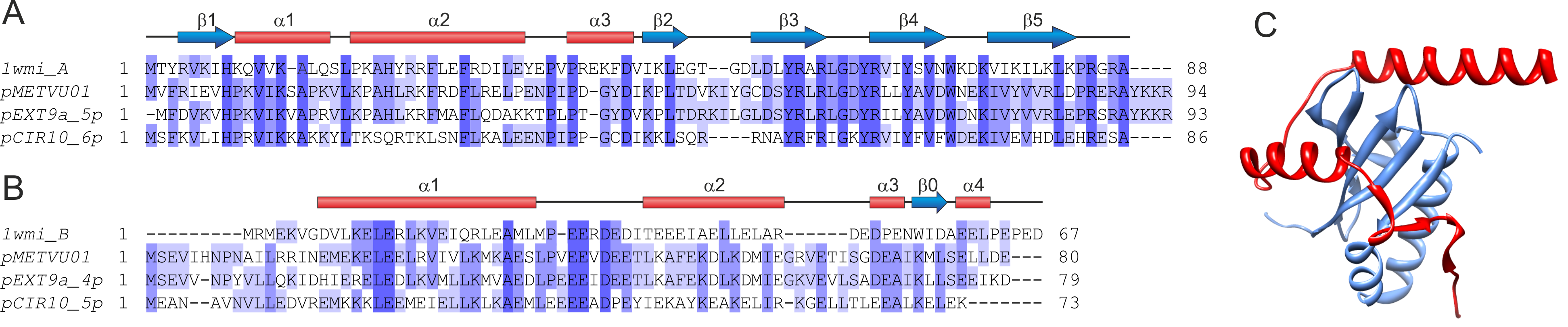

Supplement: Figure S6 — RelEB toxin-antitoxin system encoded by archaeal plasmids. Plasmid-encoded RelE-like toxin (A) and RelB-like antitoxin (B) proteins are aligned with the corresponding protein from Pyrococcus horikoshii OT3 (PDB ID: 1WMI_A and 1WMI_B, respectively). The alignments are coloured according to sequence conservation (% identity). The secondary structure determined from the X-ray structures of corresponding P. horikoshii OT3 proteins is shown above the alignment with α helices and β strands represented by red rectangles and blue arrows, respectively. (C) The X-ray structure of the RelEB complex, with RelB and RelE shown in red and blue, respectively. (TIF) [file pone.0049044.s006.tif]

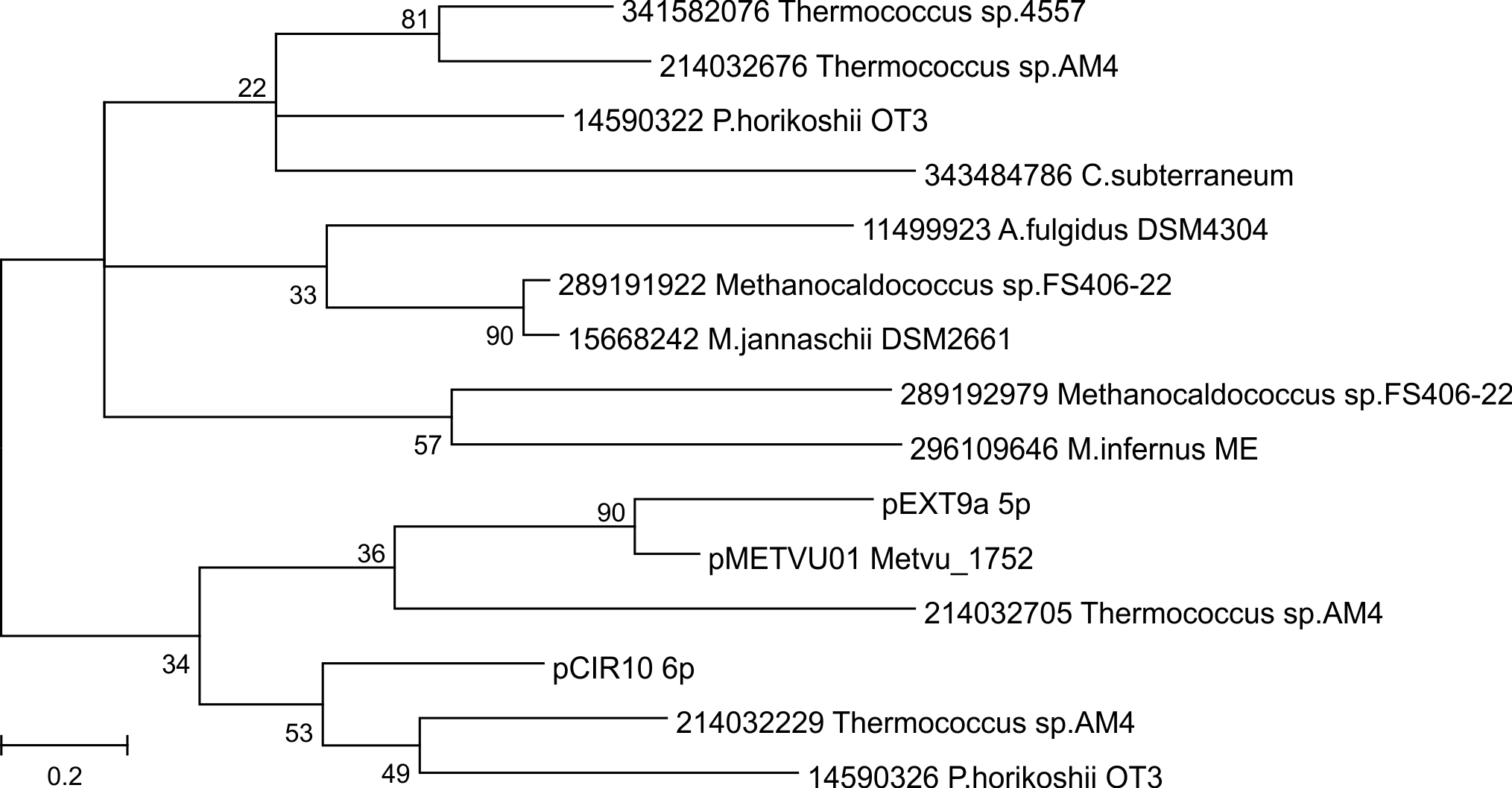

Supplement: Figure S7 — Phylogenetic analysis of the cellular and plasmid-encoded RelE-like proteins. The evolutionary history was inferred by using the Maximum Likelihood method based on the JTT model (+I, +G [4 categories]). The bootstrap consensus tree was inferred from 100 replicates. Numbers at the branch-points represent bootstrap values. Branches corresponding to partitions reproduced in less than 50% bootstrap replicates are collapsed. The scale bar represents the number of substitutions per site. The analysis involved 12 amino acid sequences. All positions containing gaps and missing data were eliminated. There were a total of 83 positions in the final dataset. Cellular RelE-like proteins are indicated by their GenBank identifiers followed by the organisms' names. (TIF) [file pone.0049044.s007.tif]

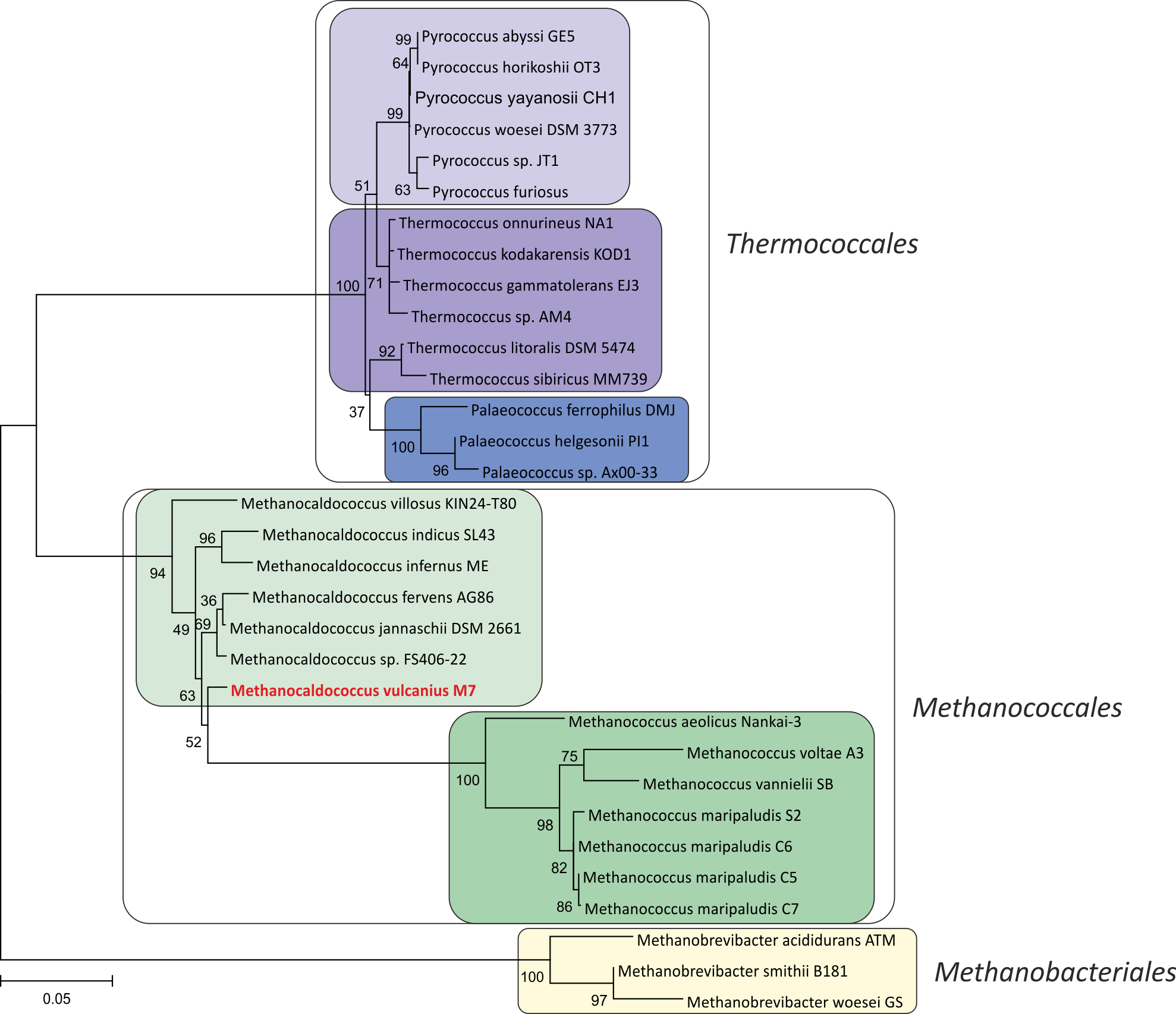

Supplement: Figure S8 — Phylogenetic tree of selected archaeal species based on 16S rRNA gene sequences. The evolutionary history was inferred by using the Maximum Likelihood method based on the Tamura-Nei model (+I, +G [4 categories]). The bootstrap consensus tree was inferred from 100 replicates. Numbers at the branch-points represent bootstrap values. Branches corresponding to partitions reproduced in less than 50% bootstrap replicates are collapsed. The scale bar represents the number of substitutions per site. The tree was rooted by using sequences of organisms belonging to the archaeal order Methanobacteriales as an outgroup. Methanocaldococcus vulcanius M7 is highlighted in red. The analysis involved 32 nucleotide sequences. All positions containing gaps and missing data were eliminated. There were a total of 1183 positions in the final dataset. (TIF) [file pone.0049044.s008.tif]
