# Supplementary material for: Insights into Dynamics of Mobile Genetic Elements in Hyperthermophilic Environments from Five New Thermococcus Plasmids
Source: PLoS One. 2013 Jan 11;8(1):e49044. doi: 10.1371/journal.pone.0049044 (PMC3543421; doi:10.1371/journal.pone.0049044)
Supplement: Materials and Methods S1 — Supporting Materials and Methods. (DOC) [file pone.0049044.s009.doc]

**SUPPORTING MATERIALS AND METHODS**

**Origin and cultivation of the new *Thermococcus* strains**

*Thermococcus* sp. AMT7 originates from a rock sample collected with the man-operated submersible *Nautile* at the top of a black smoker chimney in the East Pacific Rise (EPR), on the so-called 13°N at the Pulsar site (12°45’16’’N, 103°59’20’’W) located at 2330 m depth, during the oceanic cruise AMISTAD in June 1999. *Thermococcus* sp. EXT9 also originate from a black smoker sample collected by the submersible *Alvin* in December 2001 at the Biovent site (09°50.78' N, 104°17.58' W) at 2508 m depth, in the EPR. *Thermococcus* *sp*. IRI33 and *Thermococcus* sp. IRI48 both originate from a sample of the outer part of venting black smoker chimney of the Rainbow site (36°14’06’’N, 33°56’30’’E) on the Mid-Atlantic Ridge, at 2274 m depth. The sample was collected with the submersible *Nautile* during the IRIS oceanic cruise in June 2002. *Thermococcus sp*. CIR10 was isolated using the submersible *Alvin* from a sample of a black smoker chimney collected at the Indian Ocean ridge-ridge-ridge triple junction 25°N/70°E at 2420 m depth. All these black smoker samples were brought up to the surface in sterile and hermetic sampling boxes. Once on board, chimney samples were immediately crushed under sterile conditions in an anaerobic chamber and then immediately distributed into 100 ml serum bottles containing artificial seawater, reduced with Na_2_S. About 0.5 to 1 ml of these rock suspensions were rapidly transferred for initial enrichment into 50 ml serum bottles containing 20 ml of Thermococcales Rich Medium (TRM) that had the following composition (per liter distilled water): 3.3 g PIPES disodium salt, 23 g NaCl, 3 g MgCl_2_x6H_2_O, 0.7 g KCl, 0.5 g (NH_4_)_2_SO_4_, 1 ml KH_2_PO_4_ 5%, 1 ml K_2_HPO_4_ 5%, 1 ml Na_2_WO_4_ 10 mM, 1 ml FeCl_3_ 25 mM, 1 g yeast extract, 4g tryptone and 1 mg resazurin. The medium was adjusted to pH 6.8, autoclaved and dispensed anaerobically into sterile 50 ml serum bottles containing 1% (w/v) of steam sterilized elemental sulfur. Once the flask was hermetically closed with rubber stoppers and aluminium seals, anaerobiosis was achieved by applying cycle of vacuum and N_2_ gas to the headspace. The medium was finally reduced by injection of sterile Na_2_S solution at 0.03% final concentration. Turbidity due to microbial growth in enrichment cultures became visible after 12 h to 48 h incubation at 85°C. Dilutions of these cultures served as inoculum for plating on TRM plus colloidal-sufur plates as previously detailed . Pure cultures of strains AMT7, EXT9, IRI33, IRI48 and CIR10 were obtained from single colonies picked up from TRM plates and purified by three rounds of streaking. All these *Thermococcus spp.* strains were routinely grown overnight at 85°C under anaerobic conditions with continuous shaking (200 rpm) in 100 mL serum bottles containing 50 mL TRM. Cell growth was estimated by direct cell counting using a phase-contrast microscope and a Thoma counting chamber (0.01 mm depth; Weber, England).

**Searches for plasmid copies integrated into the host chromosomes**

Detection of putative plasmid integrated copies in the chromosome of their carrier strain were performed by Southern hybridization of total DNAs of *Thermococcus spp.* AMT7, EXT9, IRI33, IRI48 and CIR10 with their respective plasmid specific probe. Total DNA of each strain was prepared as previously described , digested by HindIII or EcoRI, separated by 0.8% agarose gel electrophoresis and transferred on a Hybond N+ membrane. About 500 ng of each plasmid DNA was digested with HindIII plus EcoRI and the mix of fragments was used to generate a probe labeled with alkaline phosphatase by AlkPhos Direct System kit (GE Healthcare, UK). Probe hybridization was carried out overnight at 42°C. After washing, the probe hybridization was detected by enhanced chemiluminescence reaction with ECL Kit (GE, Healthcare) after exposure of the membrane blot to Hyper ECL film (GE, Healthcare).

**References**

1. Erauso G, Godfroy A, Raguenes G, Prieur D (1995) Plate cultivation techniques for strictly anaerobic, thermophilic, sulfur-metabolising archaea. In: Robb FT, editor. Thermophiles, archaea: a laboratory manual. Cold Spring Harbor, N.Y.: Cold Spring Harbor Laboratory. pp. 25–29.

2. Lepage E, Marguet E, Geslin C, Matte-Tailliez O, Zillig W, et al. (2004) Molecular diversity of new Thermococcales isolates from a single area of hydrothermal deep-sea vents as revealed by randomly amplified polymorphic DNA fingerprinting and 16S rRNA gene sequence analysis. Appl Environ Microbiol 70: 1277-1286.

3. Geslin C, Le Romancer M, Erauso G, Gaillard M, Perrot G, et al. (2003) PAV1, the first virus-like particle isolated from a hyperthermophilic euryarchaeote, "Pyrococcus abyssi". J Bacteriol 185: 3888-3894.
