# Supplementary material for: Insights into Dynamics of Mobile Genetic Elements in Hyperthermophilic Environments from Five New Thermococcus Plasmids
Source: PLoS One. 2013 Jan 11;8(1):e49044. doi: 10.1371/journal.pone.0049044 (PMC3543421; doi:10.1371/journal.pone.0049044)
Supplement: Table S1 — Replication protein-based classification of Thermococcales plasmids. (DOC) [file pone.0049044.s010.doc]

Table S1. Replication protein-based classification of *Thermococcales* plasmids.

| Plasmid | Organism | Size, kb | Replication mechanism | Ref. |
| --- | --- | --- | --- | --- |
| **(i) pTN1-like** |  |  |  |  |
| **pTN1** | *T. nautilus* 30/1 | 3,6 | Rolling-circle |  |
| **pGT5** | *P. abyssi* GE5 | 3,4 | Rolling-circle |  |
| **(ii) pRT1-like*** |  |  |  |  |
| **pRT1** | *Pyrococcus* sp. JT1 | 3,4 | Unknown |  |
| **pAMT11** | *Thermococcus* sp. AMT11 | 20,5 | Unknown |  |
| **(iii) pT26-2-like** |  |  |  |  |
| **pT26-2** | *Thermococcus* sp. 26/2 | 21,6 | Theta mode |  |
| **(iv) pTBMP1-like** |  |  |  |  |
| **pTBMP1** | *T. barophilus* MP | 54,2 | Theta mode |  |
| **(v) pTN2-like** |  |  |  |  |
| **pTN2** | *T. nautilus* 30/1 | 13,0 | Theta mode |  |
| **pP12-1** | *Pyrococcus* sp. 12/1 | 12,2 | Theta mode |  |

* - Plasmids pRT1 and pAMT11 share a single open reading frame (ORF) for a putative replication protein. In contrast, 16 pRT1 ORFs are shared with an integrating element TKV1 of *T. kodakaraensis* KOD1 . Thus, replication protein-based classification does not appear to be justified in the case of pRT1 and pAMT11; the two plasmids are more likely to represent two independent families.

**References**

1. Soler N, Justome A, Quevillon-Cheruel S, Lorieux F, Le Cam E, et al. (2007) The rolling-circle plasmid pTN1 from the hyperthermophilic archaeon Thermococcus nautilus. Mol Microbiol 66: 357-370.

2. Erauso G, Marsin S, Benbouzid-Rollet N, Baucher MF, Barbeyron T, et al. (1996) Sequence of plasmid pGT5 from the archaeon Pyrococcus abyssi: evidence for rolling-circle replication in a hyperthermophile. J Bacteriol 178: 3232-3237.

3. Marsin S, Forterre P (1998) A rolling circle replication initiator protein with a nucleotidyl-transferase activity encoded by the plasmid pGT5 from the hyperthermophilic archaeon Pyrococcus abyssi. Mol Microbiol 27: 1183-1192.

4. Ward DE, Revet IM, Nandakumar R, Tuttle JH, de Vos WM, et al. (2002) Characterization of plasmid pRT1 from Pyrococcus sp. strain JT1. J Bacteriol 184: 2561-2566.

5. Gonnet M, Erauso G, Prieur D, Le Romancer M (2011) pAMT11, a novel plasmid isolated from a Thermococcus sp. strain closely related to the virus-like integrated element TKV1 of the Thermococcus kodakaraensis genome. Res Microbiol 162: 132-143.

6. Soler N, Marguet E, Cortez D, Desnoues N, Keller J, et al. (2010) Two novel families of plasmids from hyperthermophilic archaea encoding new families of replication proteins. Nucleic Acids Res 38: 5088-5104.

7. Vannier P, Marteinsson VT, Fridjonsson OH, Oger P, Jebbar M (2011) Complete genome sequence of the hyperthermophilic, piezophilic, heterotrophic, and carboxydotrophic archaeon Thermococcus barophilus MP. J Bacteriol 193: 1481-1482.
