# Supplementary material for: Insights into Dynamics of Mobile Genetic Elements in Hyperthermophilic Environments from Five New Thermococcus Plasmids
Source: PLoS One. 2013 Jan 11;8(1):e49044. doi: 10.1371/journal.pone.0049044 (PMC3543421; doi:10.1371/journal.pone.0049044)
Supplement: Table S2 — Annotation of the five plasmids described in this study. (DOC) [file pone.0049044.s011.doc]

**Table S2.** Annotation of the five plasmids described in this study.

| **pIRI33**  CDS | Protein  size (AA) | Position  (start-stop) | RBS and start codon | Putative Promoter/ Terminator | Closest Homologue  Identity ; e-value | Homologue  in pTN2 | Homologue  in pP12-1 | Conserved Motifs/Domains and predicted function(s) |
| --- | --- | --- | --- | --- | --- | --- | --- | --- |
| i33-1 | 591 | 1-1773 | ggggtggggcctaATG |  | *Mc. vulcanius* 1p (572/591) 97% ; 0 | 1p (231/598) 39% | 1p (219/590)  37% | SFI UvrD superfamily helicase (PF00580) |
| i33-2 | 51 | 1812-1964 | ggggaataATG | (+24) TTTTTCTTTAACTTT |  |  |  |  |
| i33-3 | 56 | 2143-2259 | gggggtagtATG | AAAATATATAAA (-33) |  |  |  |  |
| i33-4 | 106 | 2193-2510 | ggtggtggtattATG | AAAATATATAAA (-87)/  TTTTTCTC (+1) |  |  |  |  |
| i33-5 (-) | 85 | 2552-2806 | ggtgATG | NF/(+21) TTCCCTT | *Mc. vulcanius* 3p (55/85) 65% ; 4e-13 | 3p (35/89)  39% | 14p (25/52)  48% | wHTH, ArsR-type ; HHpred proba=99.76, e=1.3e-17; transcriptional regulator; other homologues have an N_term coiled-coil domain |
| i33-6 (-) | 78 | 3072-3305 | gacgcaaaaaaTTG | AAAGAGTTAAT (-55)/NF | *Arc. veneficus* SNP6, YP_004341572  (37/77 ; 48%) 2.7e-22 |  |  |  |
| i33-7 (-) | 185 | 3418-3972 | ggagggacgctaATG | AACTATAATAAA (-36) /CTCTTCC (+2) |  |  |  |  |
| i33-8 (-) | 180 | 4077-4616 | ggaggtgttggctgATG | TTCTTTT(+3) | *Pc. abyssi* GE5, NP_126594; 58/95 (61%); 3e-27  Mc. jannaschii DSM 2661, NP_248690; 57/162 (35%) ; 1e-13 |  |  | PIN domain containing protein VapC; possible toxin component of a TA system (TIGR00305). HHpred: Probab=99.31, E=5.1e-13, hit to 2fe1; FFAS03: score -27.1, hit to 2fe1 |
| i33-9 (-) | 142 | 4498-4923 | ggagaattgaaattggATG | AAAAAGTTTTTAAA (-56) | *Pc. abyssi* GE5, NP_126595; 61/126 (48%); 7e-19  *Mc. jannaschii* DSM 2661, NP_248689 |  |  | RHH domain antitoxin VapB;  HHpred: Probab=94.17, E=0.061, hit to 2wvb; FFAS03: score -27.5, hit to 2wvb |
| i33-10 | 178 | 5357-5890 | gagtggtggctATG |  | *Pyroccocus* sp. 12-1 plasmid p12-1, 7p;  *Tc. gammatolerans* EJ3 integrated element TGV1, YP_0029590041; 44/155 (28%);  Pc. abyssi virus1, YP_001294057; 35/141 (25%) |  | 7p (63/182) 35% | C-terminal wHTH;  HHpred: Probab=94.58, E=0.17, hit to 2xig |
| i33-11 | 62 | 5904-6089 | ggtgggtccgATG |  |  |  |  |  |
| i33-12 | 395 | 6089-7273 | ggggtgaggtcttcgaATG |  | N-term domain similar to CpB0576 of *Chlamydophila pneumoniae* TW-183, NP_876848; 30/94 (32%); 0.2 |  |  | N-terminal TMDs, central coiled-coil domain, C-terminal wHTH domain (HHpred: Probab=97.96 E=1.9e-05, hit to 1sfx) |
| i33-13 | 44 | 7339-7470 | ggcgggggagGTG |  |  |  |  |  |
| i33-14 (-) | 87 | 8241-8501 | ATG |  |  |  |  |  |
| i33-15 | 175 | 8500-9024 | gatggtgcacgccccATG | AAGGTATATATA (-35) | *Candidatus* Kor cryptofilum OPF8 YP_001737147  17/35 (49%) ; 5e-02 |  |  | N-terminal RHH domain, transcriptional regulator, HHpred: Probab=95.16 E=0.018, hit to 2hza |
| i33-16 | 671 | 9034-5 | gaggtgatcctcaATG |  | *Mc. vulcanius* 13p (616/672) 92% ; 0 | C-term of 12p (25/473) 26% | C-term of 17p (79/254) 31% | Replication protein; C-terminal wHTH domain (position 598-665), HHpred Probab=92.02, E=0.13, hit to 3ech |

| **pCIR10** CDS | Protein size (AA) | Position  (start-stop) | RBS and start codon | Putative promoter/ terminator | Closest Homologue  Identity ; e-value | Homologue  in pTN2 | Homologue  in pP12-1 | Conserved Motifs/Domains and predicted function(s) |
| --- | --- | --- | --- | --- | --- | --- | --- | --- |
| c10-1 | 568 | 1-1707 | ggagaggcATG |  | *Pyrococcus sp*. 12/1, YP_003603439; 460/567 (81%) ; 0 |  |  | SFI UvrD superfamily helicase (PF00580) |
| c10-2 | 169 | 1720-229 | ggagtggaATG |  | *Pyrococcus sp*. 12/1, YP_003603440;  *Mc. vulcanius* M7, YP_003248094, (70/169; 41%) 3e-31  *Mc. jannaschii* DSM 2661 plasmid large ECE, NP_044153 (49/165 ; 30%) 5e-12 |  | 2p (79/179 ; 44%)  9e-35 | N-term (position 12-47) Zn finger domain, HHpred: Probab=96.65, E=7e-4, hit to 2d9h; C-term (position 112-165) HFQ/SM-like RNA-binding domain;  HHpred: Probab=99.87, E=1.6e-22, hit to 1kq1;FFAS03: score -17.7 to 3hfn |
| c10-3 | 975 | 2189-5149 | ggagGTG |  | *Tc. nautilus* pTN2, YP_003603583;  *Pc. abyssi* virus 1, YP_001294073 (304/938 ; 32%) 3e-99 | 2p (624/936 ; 67%) 0 |  | Coiled-coil domain |
| c10-4 | 173 | 5207-5728 | ggtgttgcctaATG | TTATGTCTTTCC (+31) | *Tc. nautilus* pTN2, YP_003603584;  *Tc. barophilus* MP, ADT85199 (52/141 ; 37%) 3e-06 | 3p (122/172 ; 71%) 7e-66 | 14p (87/171 ;51%) | N-term coiled-coil domain;  C-term wHTH; transcriptional regulator;  HHpred: Probab=98.96 E=1.3e-09, hit to 3cuo; FFAS03: score -9.7 to 2vxz |
| c10-5 | 73 | 5819-6040 | ggtgaggatttATG | AAAAGACATATTAA (-31) | *Mc. vulcanius* M7, YP_003248083 (26/66;39%) 2e-04  *Tc. gammatolerans* EJ3, YP_002959706 (34/69 ; 49%) 1e-02 |  |  | plasmid stabilization system, antitoxin protein RelB; HHpred: Probab=90.75 E=0.15, hit to 1wmi_B |
| c10-6 | 86 | 6037-6297 | ggagttagaaaaATG | TTTTT (+15) | *Thermococcus* sp. AM4, ZP_04880209; 48/91 (53%) 3e-17 . *Tc. gammatolerans* EJ3, YP_002959705 27/55 (49%) 3e-09 |  |  | plasmid stabilization system, RelE/StbE family, protein RelE; HHpred: Probab=99.96 E=4.6e-30, hit to 1wmi_A;  FFAS03: score -46.3 to 2khe |
| c10-7 (-) | 118 | 6482-6838 | ATG | NF /TTTTTT (+63) |  |  |  |  |
| c10-8 | 177 | 7067-7600 | gagtgatgacaATG | AAAAACTTAAA (-35) | *Pyrococcus* sp. 12/1, YP_003603445 (64/181; 35%) 6e-18  *Tc*. *gammatolerans* EJ3 integrating element TGV1, YP_002959041 (54/171; 32%) 1e-05 |  |  | C-term wHTH;  HHpred: Probab=90.34, E=1.3, hit to 3eqx |
| c10-9 | 100 | 7611-7913 | gaggtgatggagATG |  | *Tc. nautilus* pTN2, YP_003603586 | 5p (36/82 ; 44%) 6e-10 |  |  |
| c10-10 | 104 | 7959-8273 | ggagggaccgatATG |  | *Tc. nautilus* pTN2, YP_003603586 | 5p (35/92 ; 38%) 2e-11 |  |  |
| c10-11 | 71 | 8201-8416 | gggtATG |  |  |  |  |  |
| c10-12 | 56 | 8496-8666 | GTG |  |  |  |  |  |
| c10-13(-) | 71 | 8826-9041 | ATG | NF |  |  |  |  |
| c10-14 | 202 | 9489-10097 | gaggtgtggaaaaaATG | AAAGGTTTATATA (-58) | *Tc. kodakarensis* KOD1, YP_184228 (14/39; 36%) 3e-02  *Arthrobacte*r sp. FB24, YP_832960 (31/121; 26%) 0.35 |  |  | N-term RHH domain, CopG/RepA family; transcriptional regulator;  HHpred: Probab=91.97, E=0.21, hit to 2wvf |
| c10-15 | 149 | 10094-10543 | ggaggtgactcctcATG |  | *Tc. nautilus* pTN2, YP_003603592 | 11p (35/118; 30%) 2e-8 | 16p (38/123; 31%) |  |
| c10-16 | 928 | 10540-1 | ggggtgagagcATG |  | *Pyrococcus sp*. 12/1, YP_003603455 | 12p (531/93 ; 57%) | 17p (646/920  70% 0 | Primase/polymerase |

| **pAMT7** CDS | Protein size (AA) | Position  (start-stop) | RBS and start codon | Putative Promoter/ Terminator | Closest Homologue  Identity ; e-value | Homologue  in pTN2 | Homologue  in pP12-1 | Conserved Motifs/Domains and predicted function(s) |
| --- | --- | --- | --- | --- | --- | --- | --- | --- |
| a7-1 | 591 | 1-1776 | gggtggggcctaGTG | TTTTTCTCC (+3) | *Mc. vulcanius* M7, YP_003248093 (574/591 ; 97%) 0 | 1p (232/598; 39%) | 1p (226/592; 38%) | SFI UvrD superfamily helicase (PF00580) |
| a7-2 | 155 | 1815-2282 | ggtggaataaTTG | AAGCCTATTATA (-27) | *Mc. vulcanius* M7, YP_003248095 (97/169 ; 57%) 2e-28 | 3p (51/163; 31%) | 14p (56/170; 33%) | Nterminal coiled-coil domain ;  C-terminal wHTH, ArsR-type; transcriptional regulator; HHpred: Probab=99.76, E=1.3e-17, hit to 1r1u |
| a7-3 | 111 | 2532-2867 | ggtgaataacATG | AAAGAAGGGATATAAA (-31) | *Staphylococcus siphovirus* 29, YP_240568 (25/72 ; 35%) 0.23 |  |  |  |
| a7-4 | 35 | 3073-3177 | ggaggtttaggcATG | AACTTAGAAACAA (-37)/ TTTTTTCCCT (+17) | *Mc. vulcanius* M7, YP_003248085 (29/35 ; 83%) 7e-13 |  |  |  |
| a7-5 | 107 | 3177-3500 | gggagagggccacaATG |  | *Mc. vulcanius* M7, YP_003248086 (102/107 ; 95%) 5e-51 |  |  | wHTH, GntR-type; transcriptional regulator; HHpred: Probab=99.12, E=1.5e-10, hit to 2y75 |
| a7-6 | 141 | 3511-3936 | gaggtgataaaaATG |  | *Mc. vulcanius* M7, YP_003248087 (69/130 ; 53%) 3e-30 |  |  |  |
| a7-7 | 119 | 3933-4292 | gagggagaggacgaATG | CCTTCCCCC (+8) | *Mc. vulcanius* M7, YP_003248088 (104/121 ; 86%) 7e-52 |  |  |  |
| a7-8 | 261 | 4620-5405 | gggggtaaaaacATG | AAACGATACCTATTAATA (-49) /CTCTCCTTTTTT (+13) | *Mc. vulcanius* M7, YP_003248089 (216/257 ; 84%) 2e-115 |  |  |  |
| a7-9 | 79 | 6214-6453 | ggatcagagaGTG | AACAGAAAGAAAA (-30) | *Mc. vulcanius* M7, YP_003248090 (71/79 ; 90%) 4e-33 |  |  |  |
| a7-10 | 45 | 6450-6587 | ggggtaaagataaaATG |  | Mc. vulcanius M7, YP_003248091 (25/47 ; 53%) 0.21 |  |  |  |
| a7-11 | 673 | 6563-8 | ggaggtgtccatATG |  | Mc. vulcanius M7, YP_003248092 (572/673 ; 85%) 0 | C-term of 12p (128/473;27%) | C-term of 17p (118/412;29% | Replication protein; C-terminal wHTH domain (position 599-673), HHpred Probab=94.19, E=0.04, hit to 3ech |

| **pIRI48** CDS | Protein size (AA) | Position  (start-stop) | RBS and start codon | Putative Promoter/ Terminator | Closest Homologue  Identity ; e-value | Homologue  in pTN2 | Homologue  in pP12-1 | Conserved Motifs/Domains and predicted function(s) |
| --- | --- | --- | --- | --- | --- | --- | --- | --- |
| i48-1 | 547 | 1-1644 | ggtgggacgATG |  | *Arc. profundus* DSM 5631, YP_003399867 (153/534 ; 29%) 3e-42 | 1p (161/570 ; 28%) | 1p (168/586 ; 29%) | SFI UvrD superfamily helicase (PF00580) |
| i48-2 | 110 | 1645-1977 | ggagggttgtgaATG |  | *Mc. vulcanius* M7 plasmid pMETVU01, YP_003248094 (44/93 ; 47%) 1e-19  *Mc. jannaschii* DSM 2661 plasmid large ECE, NP_044153 (20/52; 38%) 1e-05.  *Mc. jannaschii* DSM 2661 plasmid small ECE, NP_044176 (20/57 (35%) 4e-03 (2^nd^ iter) |  | 2p (45/89; 51%) | C-terminal (position 53-106) HFQ/SM-like RNA-binding domain; HHpred: Probab=99.90, E=7e-24, hit to 1kq1;  FFAS03: score -16.7 to 3hfn.  Other orthologues possess an N-terminal Zn finger domain. |
| i48-3 | 963 | 1990-4881 | gaggggggattagtATG | TTTCATTCTTT (+16) | *Tc. nautilus* plasmid pTN2, YP_003603583;  *Pc. abyssi* virus 1, YP_001294073 (300/947; 32%) 4e-93 | 2p (636/981; 65%) 0 |  | Coiled-coil domain |
| i48-4 | 177 | 5112-5645 | ggtgggaggtATG | NF/TTCCCCTCTTCT (+33) ? | *Tc. nautilus* plasmid pTN2, YP_003603584 | 3p (146/174 ; 84%) 2e-59 | 14p (80/173 ; 46%) | Nterminal coiled-coil domain ;  C-terminal wHTH, ArsR-type; transcriptional regulator; HHpred: Probab=99.84, E=5.7e-21, hit to 3pqk |
| i48-5 (-) | 47 | 5635-5778 | gaggGTG | NF |  |  |  |  |
| i48-6 | 129 | 5880-6269 | ggggtgtgtcataattATG |  | *Tc. nautilus* plasmid pTN2, YP_003603585;  *Pc. abyssi* virus 1, YP_001294068 (22/79 ; 28%) 1e-02 | 4p (115/129 ; 89%) 5e-62 |  |  |
| i48-7 | 107 | 6273-6596 | gaggtgtgaaagATG |  | *Tc. nautilus* plasmid pTN2, YP_003603586 | 5p (39/86 ; 45%) 4e-10 |  |  |
| i48-8 | 220 | 6602-7261 | gaggtgtaaacaaaATG |  | *Tc. nautilus* pTN2, YP_003603587 | 6p (124/221 ; 56%) 2e-66 |  |  |
| i48-9 | 173 | 7268-7789 | gggaggtgtagggaATG |  | *Tc. nautilus* plasmid pTN2, YP_003603588 | 7p (118/174 ; 68%) 1e-50 | N-term of 9p (24/60 ; 40%) |  |
| i48-10 | 368 | 7861-8967 | gaggtggcgcaggATG | CTTTTCTTTC (+1) | *Tc. nautilus* plasmid pTN2, YP_003603589;  *Mc. voltae* A3, YP_003707131 (117/367 ; 32%) 7e-47  *Pc. abyssi* virus 1, YP_001294072 (119/370 ; 32%) 3e-43 | 8p (349/368 ; 95%) 0 | 10p (168/367; 46%) | ABC ATPase;  HHpred: Probab=100, E=0, hit to 2ff7 (HlyB ABC-transporter);  FFAS03: score -20.7 to PF00265 (Thymidine kinase) |
| i48-11 | 181 | 978510324 | gaggtggtgctATG | AAAATCTTTAAA (-35) | *Tc. barophilus* MP plasmid pTBMP1, ADT85236 (29/85; 34%) 4e -03 |  | 15p (29/130; 22%) | RHH domain, CopG/RepA family; transcriptional regulator;  HHpred: Probab=94.78, E=0.033, hit to 2hza |
| i48-12 | 125 | 10324-10698 | ggaggtgaccctcaGTG |  | *Tc. nautilus* plasmid pTN2, YP_003603592 | 11p (31/89 ; 35%) 5e -8 | 16p (29/71 ; 41%) |  |
| i48-13 | 755 | 10698-12965 | ggggggtgagtgtGTG |  | *Pyrococcus sp*. 12/1, YP_003603455 |  | 17p (74/290 ; 26%) 4e-10 | Primase/polymerase; similarity in Prim-pol domain (380 aa); unique central and C-term domain |

| **pEXT9a** CDS | Protein size (AA) | Position (start-stop) | RBS and start codon | Putative Promoter/ Terminator | Closest Homologue  Identity ; e-value | Homologue  in pTN2 | Homologue  in pP12-1 | Conserved Motifs/Domains and predicted function(s) |
| --- | --- | --- | --- | --- | --- | --- | --- | --- |
| e9-1 | 591 | 1-1776 | gggtggggcccaATG |  | *Mc. vulcanius* M7, YP_003248093 (547/591 ; 93%) 0 |  |  | SFI UvrD superfamily helicase (PF00580) |
| e9-2 | 112 | 1786-2124 | gaggtgagattatg | TTTCTCTTTTTT (+24) |  |  |  |  |
| e9-3 (-) | 164 | 2121-2615 | ggtgtgagtttATG | AAATGGTTATTA (-31)/CTTTGTTC (+11) | *Pyrococcus sp*. 12/1, YP_003603452 (52/176; 30%) 2e-06  *Tc. barophilus* MP plasmid pTBMP1, ADT85199 (40/145 ; 28%) 8e-04 |  |  | Nterminal coiled-coil domain;  C-terminal wHTH; transcriptional regulator; HHpred: Probab=99.53, E=8.8e-14, hit to 3pqk |
| e9-4 | 79 | 2848-3087 | ggtgagagaATG | AAACCTATTTAA (-31)/CTTTTT (+3) | *Mc. vulcanius* M7, YP_003248083 (52/76 ; 68%) 7e-20  *Tc. gammatolerans* EJ3, YP_002960283 (47/56 ; 84%) 6e-12 |  |  | plasmid stabilization system, antitoxin protein RelB; HHpred: Probab=88.79, E=0.51, hit to 1wmi_B |
| e9-5 (-) | 93 | 3084-3365 | gggggttgcatcATG |  | *Mc. vulcanius* M7, YP_003248084 (66/93 ; 71%) 5e-42  *Tc. gammatolerans* EJ3, YP_002959705 (18/55 ; 33%) 3e-03 |  |  | plasmid stabilization system, RelE/StbE family, toxin protein RelE;  HHpred: Probab=99.91, E=1e-25, hit to 1wmi_A;  FFAS03: score -47.6 to 2khe |
| e9-6 | 66 | 3352-3552 | ggatgaaccTTG |  |  |  |  |  |
| e9-7 | 80 | 3647-3889 | gaggtatagataTTG | AAAATTAAA (-61) |  |  |  |  |
| e9-8 | 49 | 3935-4084 | gggtgtccgtATG | CCCCCTCC (+10) | *Tc. barophilus* MP, YP_004071394 (18/33 ; 55%) 2e-02 |  |  |  |
| e9-9 (-) | 114 | 3978-4325 | gggagaggacgaATG |  |  |  |  |  |
| e9-10 | 107 | 4660-4983 | ggtgataggtATG |  | *Mc. vulcanius* M7, YP_003248086 (79/107 ; 74%) 6e-41 |  |  | wHTH, GntR-type; transcriptional regulator;  HHpred: Probab=98.98, E=1.3e-09, hit to 2y75 |
| e9-11 | 141 | 4994-5419 | gagcttagccATG |  | *Mc. vulcanius* M7, YP_003248087 (70/130; 54%) 3e-31 |  |  |  |
| e9-12 | 122 | 5416-5784 |  |  | *Mc. vulcanius* M7, YP_003248088 (87/122 ; 71%) 8e-42 |  |  |  |
| e9-13 | 246 | 6140-6880 |  |  | *Mc. vulcanius* M7, YP_003248089 (79/246 ; 32%) 1e-19 |  |  |  |
| e9-14 | 104 | 7031-7345 |  |  |  |  |  |  |
| e9-15 | 132 | 8053-8451 |  |  | *Tc. barophilus* MP plasmid pTBMP1, ADT85236 (22/69 ; 32%) 1.4 |  |  | RHH domain protein; best hit to the antitoxin ParD of the ParD-ParE system.  HHpred: Probab=97.02, E=0.0046, hit to 3kxe |
| e9-16 | 675 | 8537-8 |  |  | *Mc. vulcanius* M7, YP_003248092 (526/675 ; 78%) 0 | C-term of 12p (130/458; 28%) | C-term of 17p (105/370;28) | Replication protein; C-term wHTH domain (position 601-666), HHpred: Probab=93.93, E=0.16, hit to 2fna |

*Arc. Archaeoglobus; Kor. : Korarachaeum ; Mc. : Methanocaldococcus; Pc. : Pyrococcus ; Tc. : Thermococcus ;* C_term : C-terminal; N-term : N-terminal.
