# Supplementary material for: Insights into Dynamics of Mobile Genetic Elements in Hyperthermophilic Environments from Five New Thermococcus Plasmids
Source: PLoS One. 2013 Jan 11;8(1):e49044. doi: 10.1371/journal.pone.0049044 (PMC3543421; doi:10.1371/journal.pone.0049044)
Supplement: Table S3 — PAV1 ORFs with homologues in thermococcal plasmids. (DOC) [file pone.0049044.s012.doc]

Table S3. PAV1 ORFs with homologues in thermococcal plasmids.

| **PAV1 ORFs** | **Thermococcal plasmid/ORF** | **Identity; E-value** |
| --- | --- | --- |
| ORF138 | pIRI48/ORF6 | 22/79 (28%); 1e-02 |
| ORF137 | pP12-1/ORF9 | 30/129 (23%); 1e-03 |
| ORF375 | pP12-1/ORF10 | 138/368 (38%); 2e-63 |
| ORF898 | pCIR10/ORF3 | 305/943 (32%); 1e-105 |
| ORF153 | pTN2/ORF3 | 43/168 (26%); 3e-04 |
| ORF180a | pIRI33/ORF9 | 35/141 (25%); 2e-03 |
